# Coevolution of paired receptors in *Xenopus* carcinoembryonic antigen-related cell adhesion molecule families suggests appropriation as pathogen receptors

**DOI:** 10.1186/s12864-016-3279-9

**Published:** 2016-11-16

**Authors:** Wolfgang Zimmermann, Robert Kammerer

**Affiliations:** 1 Tumor Immunology Laboratory, LIFE Center, University Clinic, Ludwig-Maximilians-University, Feodor-Lynen-Str. 19, 81377 Munich, Germany; 2 Department of Urology, University Clinic, Ludwig-Maximilians-University, Marchioninistr. 15, 81377 Munich, Germany; 3 Institute of Immunology, Friedrich-Loeffler Institut, 17493 Greifswald-Insel Riems, Germany

**Keywords:** Carcinoembryonic antigen-related cell-cell adhesion molecule (CEACAM), Paired receptors, Positive selection, Allotetraploid, Xenopus, Amphibia, ITIM, ITAM, Immunoglobulin superfamily

## Abstract

**Background:**

In mammals, CEACAM1 and closely related members represent paired receptors with similar extracellular ligand-binding regions and cytoplasmic domains with opposing functions. Human CEACAM1 and CEACAM3 which have inhibitory ITIM/ITSM and activating ITAM-like motifs, respectively, in their cytoplasmic regions are such paired receptors. Various bacterial pathogens bind to CEACAM1 on epithelial and immune cells facilitating both entry into the host and down-regulation of the immune response whereas interaction with granulocyte-specific CEACAM3 leads to their uptake and destruction. It is unclear whether paired CEACAM receptors also exist in other vertebrate clades.

**Results:**

We identified more than 80 *ceacam* genes in *Xenopus tropicalis* and *X. laevis*. They consist of two subgroups containing one or two putative paired receptor pairs each. Analysis of genomic sequences of paired receptors provide evidence that their highly similar ligand binding domains were adjusted by recent gene conversion events. In contrast, selection for diversification is observed among inhibitory receptor orthologs of the two frogs which split some 60 million years ago. The allotetraploid *X. laevis* arose later by hybridization of two closely related species. Interestingly, despite the conservation of the genomic landscape surrounding the homeologous *ceacam* loci only one locus resembles the one found in *X. tropicalis*. From the second *X. laevis* locus more than 80 % of the *ceacam* genes were lost including 5 of the 6 paired receptor genes. This suggests that once the gene for one of the paired receptors is lost the remaining gene cluster degrades rapidly probably due to lack of selection pressure exerted by pathogens.

**Conclusions:**

The presence of paired receptors and selection for diversification suggests that also in amphibians CEACAM1-related inhibitory proteins are or were used as pathogen receptors.

**Electronic supplementary material:**

The online version of this article (doi:10.1186/s12864-016-3279-9) contains supplementary material, which is available to authorized users.

## Background

A number of families of cell surface receptor with very similar extracellular domains and inhibitory or activating intracellular signaling motifs have been identified in vertebrates. The best investigated families represent the KIR, Ly49, Nkpr, SIGLEC, SIRP and CEACAM families [1]. These so called paired receptors are commonly encoded in the same gene cluster and some are thought to play a role in homeostasis of the immune system by controlling activation and downregulation of immune reactions [2]. Many of the inhibitory members of paired receptors are expressed on natural killer (NK) cells where they sense major histocompatibility antigens (MHC) present on uninfected cells leading to tolerance. Loss of MHC expression frequently found in virus infected cells releases NK cell inhibition with concomitant destruction of the infected cells. Activating members of paired receptors seem to have evolved to counter common virus immune escape mechanisms in serving as decoy receptors on NK cells. They recognize virally encoded fake MHC self-molecules that are expressed by virus infected cells thus overcoming viral immune escape by NK cell activation [1].

Other paired receptors directly interact with viral or bacterial pathogens [1]. Among those are SIRPα and CEACAM1 and CEACAM3, members of the human carcinoembryonic antigen-related cell-cell adhesion molecule (CEACAM) family which have inhibitory ITIM/ITSM motifs and activating ITAM-like motifs in their cytoplasmic regions, respectively [3, 4]. A number of bacterial pathogens like pathogenic *Neisseria* (*N. gonorrhoeae*, *N. meningitis*) *Haemophilus influenzae* and *Moraxella catarrhalis* have been shown to bind to the N-terminal immunoglobulin (Ig) variable-like domain of CEACAM1 on epithelial and immune cells allowing both entry into the host by transcytosis and down-regulation of the host’s immune response by inhibiting adaptive and innate immune reactions [5–11]. Pathogens thus exploit the normal physiological function of CEACAM1 which acts as an immune inhibitory receptor on leukocytes upon homotypic or heterotypic interactions for example with other CEACAM members [7, 12]. In contrast, binding to granulocyte-specific CEACAM3 leads to uptake and destruction of these pathogens by triggering bactericidal processes [13–16]. Interestingly, phylogenetically unrelated adhesins such as opacity-associated (Opa) protein, outer membrane protein P5 and ubiquitous surface protein (UspA1) mediate interaction with the pathogen receptor CEACAM1 indicating convergent evolution [17–19].

A host-pathogen arms race involving receptors and decoy receptors with very similar adhesin-binding domains should lead to selection of pathogens with preferential binding to the inhibitory receptor and reduced binding to its decoy counterpart. Indeed, clinical isolates of *N. gonorrhoeae* from male urethra and female genital tract often express Opa proteins which bind to CEACAM1 but not to CEACAM3 [20]. The capability of *Neisseria* to randomly switch on expression of variant Opas from a panel of *Opa* genes aides natural selection from a heterogenous *Neisseria* population. On the other hand, individuals with variant CEACAM1 receptors with low or no binding to pathogens should have an selective advantage. This will inevitably lead to poorly matched paired receptors and loss of decoy function. Intrachromosomal recombination or gene conversion between exons encoding ligand-binding domains of inhibitory and activation receptors within the *CEACAM* gene cluster could correct this deficit. Indeed, replacement of part of *CEACAM3* exon 2 encoding the ligand-binding domain with sequences from the corresponding exon of *CEACAM1* has happened in humans [3, 21].

CEACAM families differ greatly in gene number and domain composition of the encoded proteins between mammalian species. Most of the analyzed mammals also contain putative paired CEACAM receptors [3, 22]. Allelic variants of CEACAM1 in mice and cattle have been shown or are suspected to serve as coronavirus receptors [23, 24]. Therefore, the rapid divergence of CEACAM1 and corresponding activating receptors during mammalian evolution is thought to be pathogen-driven [1, 3, 22].

Also more distantly related *CEACAM* genes exist in mammals (*CEACAM16, CEACAM18, CEACAM19* and *CEACAM20*) which do not represent paired receptors. They are clustered distally from the *CEACAM1*-related genes. They differ in domain organization and sequence among each other and exhibit specialized functions [25–27]. However, they are conserved between mammalian species which allows unequivocal assignment of orthologs [3].

*CEACAM* gene families seem to be restricted to vertebrates. *CEACAM* family members have been recently identified in reptiles, amphibians and in bony and cartilaginous fishes [28, 29]. However, the exact composition, the presence of paired receptors and the driving forces behind their evolution have not been investigated. Here we present comprehensive analyses of the *ceacam* families of two clawed frog species; the western clawed frog *Xenopus tropicalis* and the African clawed frog *X. laevis* the ancestors of which split some 60 million years ago [30]. We identified two distantly related *ceacam* families which both contain rapidly evolving paired receptors. Analysis of the *ceacam* family in *X. laevis* allowed us to follow the fate of a group of rapidly evolving genes after allotetraploidization.

## Results

### Identification of *ceacam* gene families in *X. tropicalis* and *X. laevis*

**Fig. 1:**
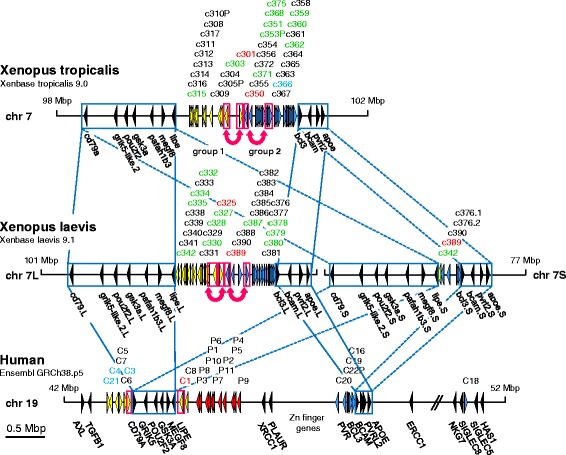
Chromosomal arrangement of *ceacam* genes in *X. tropicalis* and *X. laevis*. *Arrowheads* represent genes with their transcriptional orientation. Xenopus *ceacam* gene clusters are subdivided in group 1 (*yellow*) and group 2 members (*blue*). For *X. laevis* two homeologous *ceacam* clusters are found on chromosome 7 L and 7S which were generated by hybridization during speciation. Homeologs are indicated by L and S. Human *CEACAM1*-related genes are indicated in *yellow*, when predominantly expressed in trophoblast cells in *red*; the *CEACAM* genes for which orthologs can be identified in mammals are shown in *blue* and marker genes in *black*. Their syntenic relationship is shown by *blue* lines. Names of *CEACAM1*-like genes with ITIM-encoding exons are shown in *red* and with ITAM and ITAM-like motif-encoding exons in *green* and *blue*, respectively. *Red double arrows* symbolize potential recombination events between regions with *ceacam* genes containing ITIM or ITAM/ITAM-like motifs (genes shown in *red* boxes). Note that, in general, the genes in the same subcluster show the same, in different subclusters opposite transcriptional orientation. The nucleotide numbering of the chromosomes starts at the telomere of the short arms located to the left. The databases and the versions used are indicated below the species name. c, *ceacam,* C, *CEACAM*; chr, chromosome; P, pregnancy-specific glycoprotein (PSG) genes

**Fig. 2:**
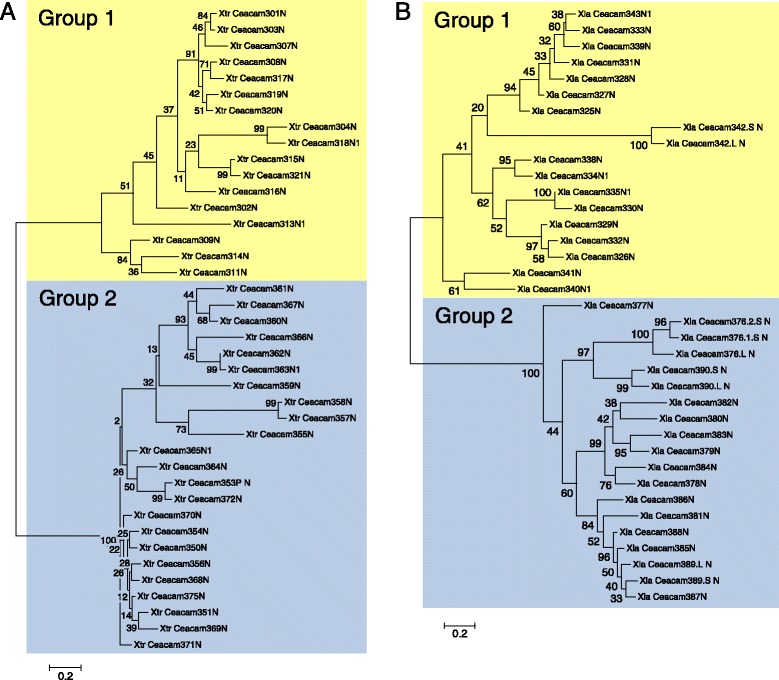
Phylogenetic relationship of *Xenopus* Ceacam proteins. Phylogenetic trees were constructed based on mature N domain amino acid sequences (signal peptides excluded) from *X. tropicalis* (**a**) and *X. laevis* Ceacams (**b**) using the Maximum Likelihood method (MEGA6 software). The tree with the highest log likelihood is shown. The percentage of trees in which the protein sequences clustered together is shown next to the branches. For Ceacams predicted to contain more than one N domain, only the most N-terminal N1 domains were included in the analysis. Two distantly related Ceacam groups of N domains termed group 1 and group 2 can be discriminated in both species. Some of the most closely related Ceacams in the allotetraploid *X. laevis* represent homeologs located on the small (S) or the large (L) version of chromosome 7. A closely related pair of paralogous proteins termed .1 and .2 is found on chromosome 7S but not on chromosome 7 L. The bar below the phylogenetic tree shows the scale for the number of substitutions per site. P, pseudogene; *Xla, X. laevis; Xtr, X. tropicalis*

Based on their syntenic location between the flanking genes *lipe* and *bcl3*, and the presence of exons with conserved phasing encoding Ig variable (IgV)- and Ig constant (IgC)-like domains and ITIM and ITAM-like motifs most similar to mammalian CEACAM members (Fig. 1 and Additional file 1) 44 and 38 *ceacam* genes were identified on chromosomes 7 in *X. tropicalis* and *X. laevis*, respectively (Additional files 2 and 3). Interestingly, two *ceacam* gene loci exist in *X. laevis* on the homeologous chromosomes 7 L and 7S generated during speciation by hybridization of closely related species (Fig. 1; for nomenclature see [31]). Amino acid sequence comparison of the N-terminal IgV-like domains (N domains) revealed the presence of two distantly related subgroups group 1 and group 2 in both species (Fig. 2). N domains were chosen because they represent functionally important domains which have been shown in other species to be responsible for ligand binding [32]. Group 1 and group 2 genes are localized in clusters next to each other and, different from mammals, are not disrupted by *non-ceacam* genes (Fig. 1). Group 1 and group 2 Ceacam N domain amino acid sequences are most closely related to cartilaginous and bony fish and reptile and mammalian CEACAM N domain sequences, respectively (~35 % identity). Within subgroup 1 and 2, members exhibit between 40 and 93 % N exon amino acid sequence identity, while between subgroups only 20-30 % sequence identity is observed (data not shown). Similarly, transmembrane and cytoplasmic sequences also exhibit a higher degree of identity within groups than between groups (Additional file 1 and data not shown). Despite the low sequence identity group 1 and group 2 Ceacam IgV-like domains exhibit a very similar three-dimensional structure predicted by modeling using corresponding human and murine CEACAM1 sequences (Additional file 4).

Taken together, this indicates that two *ceacam* groups exist in *Xenopus* which were probably derived early in amphibian evolution possibly from two different *ceacam* ancestors and their origin predates the divergence of *X. laevis* and *X. tropicalis*.

### Groups of paralogous Ceacams contain paired receptors

**Fig. 3:**
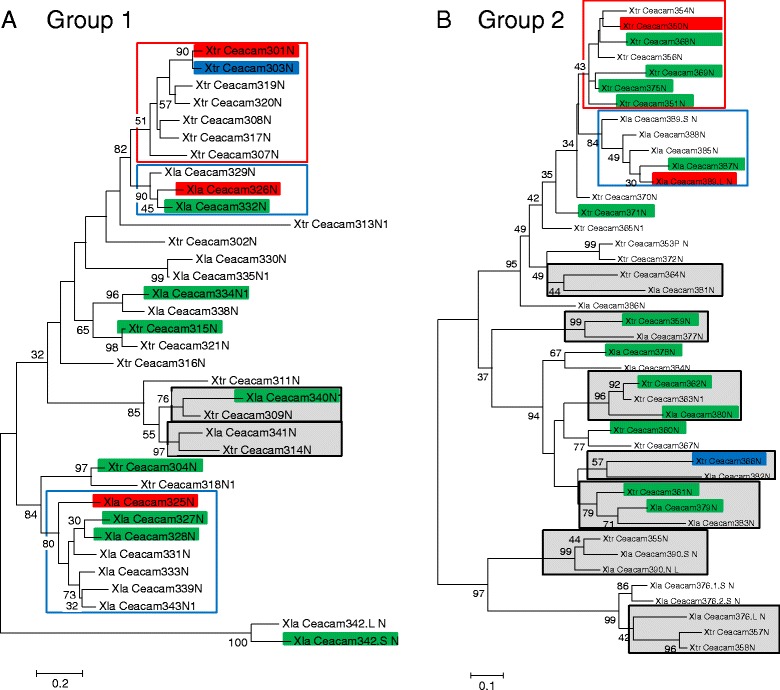
Orthologous and paralogous members of the Ceacam family in *X. tropicalis* and *X. laevis*. Phylogenetic trees were constructed based on mature N domain amino acid sequences (signal peptides excluded) from *X. tropicalis* and *X. laevis* group 1 (**a**) and group 2 Ceacams (**b**) using the Maximum Likelihood method (MEGA6 software). The tree with the highest log likelihood is shown. The percentage of trees in which the protein sequences clustered together is shown next to the branches. For Ceacams predicted to contain more than one N domain, only the most N-terminal N1 domains were included in the analysis. Ceacams whose N domains exhibit the highest degree of identity within the same species (paralogs) are boxed with *red* and *blue* lines for *X. tropicalis* and *X. laevis*, respectively. Pairs consisting of most closely related proteins from different species (sometimes including recently generated paralogs) are highlighted in *gray* (orthologs). The names of ITIM-containing members are highlighted in *red*, proteins with ITAM and ITAM-like motifs are marked with *green *and *blue* background, respectively. P, pseudogene; *Xla, X. laevis; Xtr, X. tropicalis*

**Fig. 4:**
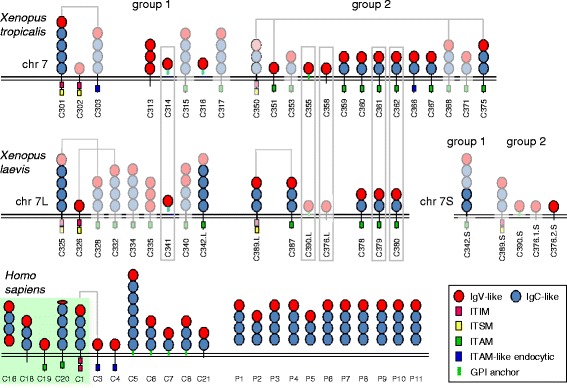
Domain organization of *Xenopus* Ceacam proteins. The domain organization of Ceacam family members from *X. tropicalis* and *X. laevis* was predicted by gene analysis only (domains shown in *light colors*) or, where available, confirmed by EST and cDNA sequences (domains shown in *intense colors*). Only Ceacams whose complete domain organization could be delineated were included. Homeologous Ceacams in *X. laevis* arisen by hybridization during speciation are discriminated by L (encoded on long chromosome variant 7) and S (encoded on short chromosome variant 7). For comparison the domain organization of human CEACAM proteins is shown. The conserved human CEACAM family members are highlighted in *green*. IgV-like domains are shown as *red*, IgC-like domains are *blue* ovals. The predicted signaling motifs in the cytoplasmic domains are schematically shown as *green* (ITAM), *blue* (ITAM-like motif),* red* (ITIM) and *yellow boxes* (ITSM). Transmembrane domains and GPI anchors are indicated by *black* and *green lines*, respectively. Orthologous relationship as suggested by sequence relatedness and/or synteny is indicated by *gray* boxes. Note that Xtr_Ceacam301 and Xla_Ceacam326 appear to represent orthologs based on the degree of N domain sequence identity (see Fig. 3) despite their different domain organization. Paired receptors identified by their similar N domain sequences and the presence of ITIM/ITSM or ITAM/ITAM-like motifs are connected by *gray* lines. C, CEACAM or Ceacam; P, PSG

In mammals, CEACAM families consist of a group of orthologous members (CEACAM16, CEACAM18, CEACAM19, CEACAM20) where counterparts can be clearly assigned in different species and a group of paralogous members which are most closely related to CEACAM1 within the same species [3]. To identify orthologous Ceacam pairs as well as Ceacam paralogs, *X. tropicalis* and *X. laevis* group 1 and group 2 amino acid sequences from mature N domains (signal peptide sequence removed) were compared and their relationship displayed as dendrograms. In group 1 and group 2, two and seven pairs of orthologous Ceacams, respectively, could be identified based on their degree of sequence identity (Fig. 3). Their predicted domain organization is heterogenous. Three members consist of only one IgV-like domain and are either secreted or membrane-bound by a transmembrane domain or a GPI anchor while two transmembrane-bound orthologous pairs are composed of one IgV- and one IgC-like domain and an ITAM-containing cytoplasmic region (Fig. 4).

In addition, sets of proteins exist whose closest relatives are found in the same species thus representing paralogous proteins: one and two in *X. tropicalis* and *X. laevis* group 1 Ceacams, respectively, and one in each species in group 2 (Fig. 3). Interestingly, these groups of closely related Ceacam members harbor one member with an ITIM and one or more with ITAM-like motifs (Fig. 3; Additional file 1). Pairs of cell surface proteins with similar extracellular domains which are able to interact with the same ligand, however, transmitting opposing i.e. inhibitory or activating signals represent so called paired receptors. Based on these definition, in *X. tropicalis* Ceacam301 and Ceacam350 with an ITIM and Ceacam303 and Ceacam351 or Ceacam368 with an ITAM-like motif and an ITAM, respectively, and correspondingly in *X. laevis* Ceacam325 and Ceacam326 (ITIM) and Ceacam327 or Ceacam328 and Ceacam332 (ITAM) and Ceacam389 (ITIM) and Ceacam387 (ITAM) could represent paired receptors (Figs. 3 and 4). These putative paired receptors share between 80 and 93 % of their IgV-like ligand binding domain amino acid sequences (data not shown).

In summary, orthologous and paralogous members exist in both Ceacam groups. Putative paired receptors could be identified among the paralogous members.

### Similarity of receptor binding domains of paired receptors is maintained by recombination

**Fig. 5:**
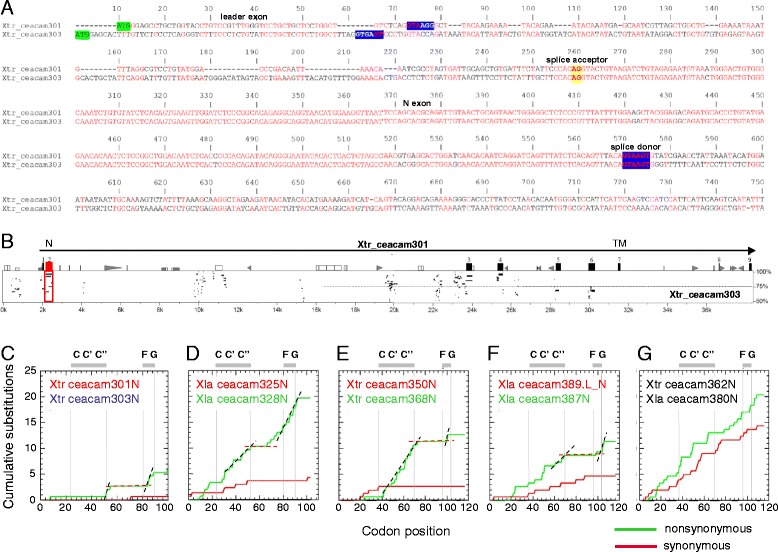
Evidence for gene conversion between putative paired receptor *ceacam* genes. (**a**) Alignment of exons 1 and 2 encoding the leader and N domain, respectively, and flanking introns of *ceacam301* and *ceacam303.* Note the strong sequence conservation restricted to the N domain exon. The start codons are marked in *green*, the splice acceptor and splice donor sequences are highlighted in *yellow* and *blue*, respectively. (**b**) The nucleotide sequence of ITIM/ITSM-encoding *ceacam301* gene was compared with that of ITAM-like motif-encoding *ceacam303* from *X. tropicalis*. For contiguous stretches of nucleotides conserved between the gene pairs using a sliding window, the degree of identity was calculated and displayed as horizontal lines. The location of *ceacam301* exons is indicated by numbered boxes. The genomic region involved in gene conversion is marked with a *red* box, the affected N exon is shown in *red*. Different repeat sequences are indicated by differently shaped forms. (**c**-**f**) The accumulation of nonsynonymous (*green curves*) and synonymous substitutions (*red curves*) along the N exons of putative paired receptor genes were determined after manual removal of gaps in the compared nucleotide sequences. The type of encoded signaling motif is indicated by the color of the gene name: *red*, ITIM/ITSM; *green*, ITAM; blue, ITAM-like motif. Stretches of codons with no or minimal accumulation of synonymous substitutions which run parallel to the x-axis suggest recent gene conversion/recombination events. Note the rapid accumulation of nonsynonymous mutations in the CC’C”FG β-strand regions (*black broken lines*) which indicates selection for diversification. This contrasts with conserved regions indicated by *red broken lines*. For comparison, N exon sequences of an orthologous gene pair (Xtr *ceacam362*/Xla *ceacam380*) were analyzed (**g**). The location of CC’C”and FG β-strand regions determined by 3D modeling (Additional file 4) are indicated by *gray* boxes above the graphs. N, N domain exon; TM, transmembrane domain exon; Xla, *X. laevis*; Xtr, *X. tropicalis*

In paired receptors, pathogen-binding regions have to stay similar thus allowing the host to counterbalance the immune suppressive signal elicited by engagement of the inhibitory receptor through the pathogen by providing an activating receptor as a mimic [33, 34]. In other paired receptor gene families this is often achieved by recombination or gene conversion between genes encoding inhibitory and activating receptors, restricted to the gene region encoding the ligand binding domains [33]. We, therefore, screened the potential *Xenopus* paired receptor genes for recombination/gene conversion events. Indeed, in *X. tropicalis* group 1 *ceacam301* and *ceacam303* and in group 2 *ceacam350* and *ceacam351*/*ceacam368* N exons have recently undergone gene conversion which is exactly restricted to the exon just including the splice consensus sequences. This is supported by the high conservation of N exon sequences with virtual absence of synonymous mutations (which in general do not encounter purifying selection) and very low sequence conservation in the flanking introns (Fig. 5a, b, c; Additional file 5). No other genomic regions seem to have been involved in the gene conversion event in *ceacam301* and *ceacam303* (Fig. 5b). Recombination events were also noticed in N domain exons of other putative paired receptor gene pairs. This was evident from the lack or low rate accumulation of synonymous mutations mostly restricted to certain regions of the N exons of receptor pairs like *ceacam325* and *ceacam328* and possibly of *ceacam389* and *ceacam387* in *X. laevis* and *ceacam350* and *ceacam36*8 in *X. tropicalis* (Fig. 5d-f). Without recurrent recombination/gene conversion events between paralogous genes one would expect a steady accumulation of synonymous nucleotide changes along the N exons as it is found between *X. tropicalis* and *X. laevis ceacam* orthologs, like *ceacam362* and *ceacam380* (Fig. 5g). Interestingly, possibly due to continued pressure from pathogen adhesin-receptor interaction, regions which represent putative interaction sites (the CC’C”FG face of the Ig fold [35]) appear to rapidly accumulate non-synonymous mutations occurring after a gene conversion event (Fig. 5c-f). For intrachromosomal recombination/gene conversion to take place, involved genes must exhibit opposite transcriptional orientation in order that homologous sequences can be aligned with looping out of the intervening sequences [3, 36]. Indeed, putative paired receptor genes in *X. laevis* and *X. tropicalis* from both *ceacam* subgroups exhibit opposite transcriptional orientation with group 1 and group 2 ITIM-encoding genes facing each other (Fig. 1).

The non-random transcriptional orientation of *ceacam* genes encoding proteins with inhibitory or activating signaling motifs, gene conversion within exons encoding ligand binding domains as well as the conservation of these domains in ITIM and ITAM-containing Ceacams strongly argue that these Ceacams function as paired receptors.

### Selection for diversification in paired receptor Ceacam groups

**Fig. 6:**
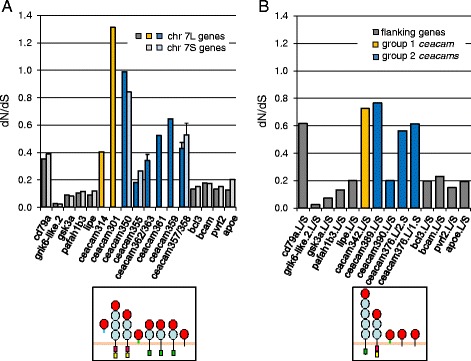
Differential conservation of orthologous and homeologous *Xenopus ceacam* and flanking genes. Nucleotide sequences of N exons and the open reading frames from *ceacam* and flanking genes, respectively, from *X. tropicalis* were compared codon-wise with each of the two homeologous orthologs of *X. laevis* (**a**) or the *X. laevis* homeologs with each other (**b**) after manual removal of gaps and the ratio of the rate of nonsynonymous and synonymous mutations was calculated. The resulting dN/dS values were plotted using the *X. tropicalis* gene order on chromosome 7 (**a**) or the *X. laevis* gene order on chromosome 7S (**b**). In cases of recent gene duplication of one of the orthologs in *X. tropicalis* or *X. laevis* dN/dS ratios for all orthologous pairs were calculated and plotted as mean and deviation or standard deviation. The type of signaling motif present in the corresponding proteins can be inferred from the schematic representation of the protein domains below the graphs (see Fig. 4 for keys to domains and motifs). Chr, chromosome

Pathogen receptors which allow entry into a host often exhibit selection for diversification of their amino acid sequences. This is evident from high ratios (>1) of their rate of non-synonymous over their rate of synonymous mutations (dN/dS) especially in regions relevant for pathogen binding [37]. When whole domains or proteins are analyzed the dN/dS ratios can drop below 1 despite the presence of regions with strong positive selection, due to the presence of regions with negative or neutral selection. To test whether ITIM/ITSM-bearing Ceacam receptors in *Xenopus* might represent pathogen receptors as found for human and mouse CEACAMs we analyzed dN/dS ratios of N domain exons of *ceacam* orthologs in *X. tropicalis* and *X. laevis*. Orthologous genes encoding receptors with ITIM exhibited the highest dN/dS ratios, i.e. 1.3 for *Xtr ceacam301/Xla ceacam326* (group 1) and 1.0 for *Xtr ceacam350/Xla ceacam389.L* orthologs (group 2) (Fig. 3; Fig. 6a). In contrast, dN/dS ratios between 0.18 and 0.65 were found for the other *ceacam* orthologs. The lowest dN/dS ratios were observed for the flanking non-*ceacam* genes (dN/dS = 0.1–0.25) with the exception of the immune function gene *cd79a* (dN/dS = 0.35), which encodes an ITAM-bearing component of the B cell receptor. The orthologous *ceacam* pair with the lowest dN/dS ratio (dN/dS = 0.18) encodes glycosylphosphatidylinositol (GPI) membrane-anchored proteins (Fig. 6a). No large dN/dS ratio differences were found when *X. tropicalis* genes were compared with the *X. laevis* homeologs on chromosome 7S (Fig. 6a) which are paralogous genes that were formed by the hybridization event in *X. laevis* which probably occurred during speciation (see below). This suggests that there is no loss of function or gain of new function for one of the two homoeologs.

N exon dN/dS ratios of ≥ 1 of *Xenopus* inhibitory receptor orthologs indicate selections for diversification typically observed in pathogen receptors.

### Selective gene loss in *ceacam* locus on chromosome 7S created by allotetraploidization in *X. laevis*

In *X. laevis,* allotetraploidy was probably caused by hybridization during speciation which led to whole genome duplication including the *ceacam* gene locus on chromosome 7. Despite the overall conservation of the duplicated genomic region surrounding the *ceacam* loci only the locus on chromosome 7 L resembles that found in *X. tropicalis*. More than 80 % of *ceacam* genes were lost from the locus on chromosome 7S and only 1 and 3 genes are left from group 1 and group 2 genes, respectively (Fig. 1). Interestingly, no paired receptor pair is preserved at the 7S locus; only the group 2 inhibitory receptor gene (*ceacam389.S*) is retained. Notwithstanding the massive *ceacam* gene loss, a gene duplication event probably after *X. laevis* speciation led to the generation of two closely related *ceacam376* genes (*ceacam376.1.S* and *ceacacam376.2.S*) next to the *bcl3* gene (Fig. 1).

**Fig. 7:**
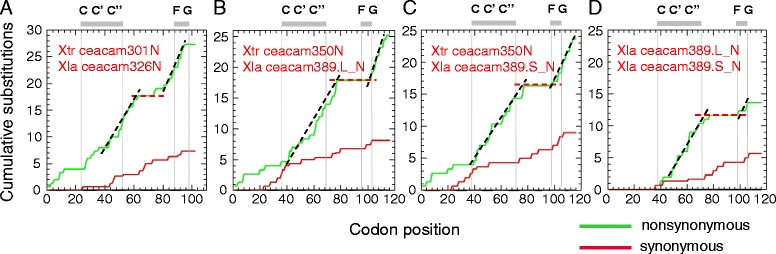
Selection for sequence diversification in inhibitory receptor Ceacams. The accumulation of non-synonymous (*green curves*) and synonymous substitutions (*red curves*) along the N exons of orthologous (**a**-**c**) and homeologous inhibitory receptor gene pairs (**d**) were determined after manual removal of gaps in the compared nucleotide sequences. The names of the analyzed genes are indicated in the top left corner of the graph. Note the rapid accumulation of nonsynonymous mutations in the CC’C”FG β-strand regions (*black broken lines*) which indicates selection for diversification. This contrasts with conserved regions between CC’C” and FG β-strands indicated by *red broken lines*. The location of CC’C” and FG β-strand regions determined by 3D modeling (Additional file 4) are indicated by *gray* boxes above the graphs. Note the sequence identity of the 38 N terminal codons in the N exon of *X. laevis* homeologs *ceacam389.L* and *ceacam389.S* possibly caused by interchromosomal recombination. N, N domain exon; Xla, *X. laevis*; Xtr, *X. tropicalis*

To determine whether selection occurs to maintain the function of both homeologous gene copies, dN/dS ratios for N domain exons or whole coding sequences were determined for *ceacam* and flanking genes, respectively. Only one *ceacam* gene which encodes a GPI-linked group 2 member reveals similar conservation with a dN/dS ratio of 0.2 as flanking genes which exhibit dN/dS ratios between 0.03 and 0.2 with the exception of *cd79a* exhibiting a dN/dS ratio of 0.6 (Fig. 6b). In addition, both copies of all homeologous genes exhibit similar dN/dS ratios when compared with the *X. tropicalis* orthologs (Fig. 6a). Therefore, both homeologous copies of *ceacam390.L*/*390.S* and the non-*ceacam* genes seem to be functional and under selective pressure. In contrast, much higher dN/dS ratios between 0.6 and 0.75 are observed for the remaining *ceacam* genes, indicating either lack of selection for conservation of the homeologous pairs or selection for diversity. The latter appears to be at work for the inhibitory receptor-encoding genes *ceacam389.L*/*389.S* which exhibit the highest dN/dS ratios (Fig. 6a, b). In addition, regions with increased accumulation of non-synonymous mutations which are similar to those found for orthologous or homeologous pairs of inhibitory receptors could be identified. They co-localize with the CC’C”FG β-sheet which represent putative pathogen binding sites (Fig. 7).

## Discussion

### Amphibian *ceacam* gene family exhibits an ancestral genomic arrangement

In this comprehensive analysis *ceacam* genes could be identified in *X. tropicalis* and *X. laevis* based on synteny and structural homology despite a low degree of sequence identity which is due to rapid divergence during evolution. The *ceacam* gene family consists of two distantly related subgroups (group 1 and group 2) with 15–20 members each. Only few orthologous *ceacam* members (mostly in group 2) were found in the two frog species. The *Xenopus ceacam* genes are arranged in one cluster separated by subgroups uninterrupted by non-*ceacam* genes (Fig. 1). This is also observed for most of the *CEACAM* genes in the marsupial opossum (*Monodelphis domestica*) but not in eutherian mammals. Here, the *CEACAM* locus is interrupted by two large regions with non-*CEACAM* genes (Fig. 1; [3]). This indicates that a continuous *ceacam* gene cluster was also present in the last common ancestor of amphibians and mammals.

No *CEACAM16*, *CEACAM18*, *CEACAM19* and *CEACAM20* genes, which are well conserved in mammals and are clustered next to *BCL3,* were found in *Xenopus* [3]. However, *CEACAM19* orthologs can be identified unequivocally in reptiles including turtles, snakes, alligators and gecko but not the other genes ([29]; Zimmermann, unpublished results). Interestingly, group 2 *Xenopus ceacam* genes which are also located next to *bcl3* but not group 1 genes are most closely related to reptilian *CEACAM19* (32–37 % identity) representing the most common hits outside of the anuran order when *Xenopus* Ceacam group 2 N exon amino acid sequences are used as query sequences. This might indicate that *CEACAM19* in reptiles and mammals and group 2 *ceacam* genes share a common ancestor.

### Two paired receptor systems exist in the *Xenopus* Ceacam family which exhibit signs of pathogen-mediated selection

Each subgroup contains one or two paired receptors with oppositely signaling ITIM and ITAM/ITAM-like motifs and highly similar ligand binding domain (N domain) amino acid sequences (Figs. 3 and 4). This is different from mammalian CEACAMs which typically have only one set of paired receptors or none as found for mouse and rat [3].

What is the evidence that the paired Ceacam receptors are being or have been used as pathogen receptors in *Xenopus*? Both diversification of pathogen receptors to avoid binding of the pathogen (indicated by high dN/dS ratios) and maintenance of similarity of the pathogen adhesin-interacting domain in paired receptors functioning as pathogen or decoy receptors will be selected for in a pathogen/host arms race [1, 34, 37]. Indeed this is observed in both *Xenopus* Ceacam groups. The exons encoding the ligand-binding domains of *X. tropicalis* and *X. laevis* ITIM-containing orthologs exhibit the highest dN/dS ratios which is indicative of positive selection (Fig. 6a). Positively selected amino acid positions of a protein domain are expected to reside at the site of contact between the pathogen adhesin and its receptor [37]. This seems to be the case for putative Ceacam pathogen receptor orthologs in *X. tropicalis and X. laevis* which show selective accumulation of non-synonymous mutations in the CC’C” FG face of the IgV-like domain (Fig. 7a-c) responsible for pathogen interaction [35]. In contrast, the Ig β-sheet on the opposite side of the Ig fold is highly conserved. This recurrent host escape and pathogen adaptation (“Red Queen” scenario [38]) can lead to an imbalance with pathogen binding to the entry/inhibitory receptor being maintained while the decoy receptor-pathogen interaction is abolished [20]. This problem can be resolved through recombination/gene conversion whereby the decoy receptor pathogen binding domain is replaced by that of the pathogen receptor. Absence of synonymous mutations in large regions of the N exons revealed by comparison of the putative pathogen receptor and decoy receptor sequences suggest intra- or interchromosomal recombination or gene conversion events as cause of the similarity of paired receptor ligand binding domains (Fig. 5c-e). Original sequence identity is rapidly lost due to the on-going struggle between host and pathogens again noted by the selective accumulation of non-synonymous mutations in the presumed adhesin binding regions (Fig. 5c-e).

Comparative analyses of mammalian *CEACAM* loci revealed inversion of regions with non-*CEACAM* genes between oppositely oriented *CEACAM1* and presumed decoy receptors genes in some species. This suggested that recombination involved an intrachromosomal loop formation mechanism that allows alignment of the exon sequences encoding the ligand binding domains [3]. The recombination mechanism in *Xenopus* is still unclear. However inverted transcriptional orientation of ITIM- and ITAM-bearing genes indicates a similar intrachromosomal recombination mechanism.

### Loss of paired receptors from one of the two homeologous *X. laevis ceacam* loci

At the time of hybridization of two ancestral *X. laevis* species some 40 million years ago [30] which led to speciation, two probably functionally distinguishable sets of *ceacam* genes existed. The set on chromosome 7 L was better functioning presumably with respect to pathogen resistance and was consequently retained [39]. Loss of the *ceacam* locus on chromosome 7S is not complete. Interestingly, in both subgroups no paired receptor system persisted. Only one ITIM- and one ITAM-bearing Ceacam is found in group 2 and group 1, respectively (Fig. 6b). This indicates that once the genes for inhibitory or activating members have been lost the remaining gene cluster degrades rapidly probably due to lack of selection pressure exerted by pathogens which helps to maintain paired receptors.

## Conclusions and Perspectives

Although we do not know which specific pathogens bind to to Ceacam receptors in *Xenopus* the presence of closely related paired receptors as well as selection for diversification suggests that also in amphibians CEACAM1-related inhibitory proteins still are or have been exploited in the past as pathogen receptors and similar defense strategies have been developed in amphibians and mammals by convergent evolution. Thus the *CEACAM* family is a prototype gene family which offers a unique opportunity to study “arms races” caused by host/pathogen interactions.

CEACAM1-related pathogen receptors serve a dual role: They allow both entry into the host and inhibition of inflammatory responses to pathogen infections. Therefore, CEACAM1-like receptors are expected to be expressed on epithelial surfaces as well as on leukocytes involved in innate and adaptive immunity. Decoy receptors should be expressed on cells of the innate immune system which allow uptake and destruction of pathogens. The identification and characterization of individual members of the *Xenopus ceacam* gene families will now allow to use next generation sequencing data of RNA from multiple organs and cell types of *Xenopus* species as well as genomic sequence data of additional frog species like that of *Nanorana parkeri* a member of the species-rich *Neobatrachia*, which contains the vast majority of amphibian taxa (Sun et al., 2015) to support the suggested pathogen defence function of anuran *ceacam* families.

## Methods

### Identification and nomenclature of genes

Sequence similarity searches were performed using the NCBI BLAST tools (http://www.ncbi.nlm.nih.gov/BLAST) and the Ensembl BLAST/BLAT (http://www.ensembl.org/Xenopus_tropicalis/Tools/Blast?db=core) and Xenbase BLAST (http://www.xenbase.org/genomes/blast.do) search programs. For identification of *ceacam* genes regions syntenic to mammalian *CEACAM* loci were analyzed for the presence of Ig domain-encoding genes. The following databases were used for *ceacam* gene identification and loci analyses: Xenbase *X. laevis* J-Strain 9.1 and *X. tropicalis* Nigerian 9.0 (http://www.xenbase.org/entry/) and Ensemble *X. tropicalis* JGI 4.2 (http://www.ensembl.org/Xenopus_tropicalis/Info/Index). *Xenopus* genomes were reprobed with exon sequences from newly discovered *ceacam* genes. For estimation of the number of *ceacam* genes present in a given species, distinct *ceacam* N domain exons with a sequence divergence > 1 % were counted. Multiple N exons with no annotated non-N exon in between were considered to belong to the same gene. Genes that contained stop codons within their N domain exons or lacked appropriate splice acceptor and donor sites were considered to represent pseudogenes. Genes were assigned to their respective *ceacam* subgroups 1 and 2 based on phylogenetic analyses. Due to their non-orthologous relationship with mammalian CEACAM genes the new *ceacam* genes were numbered independently as follows: *X. tropicalis,* group 1: *ceacam301-ceacam321*; *X. tropicalis,* group 2: *ceacam350*-*ceacam375*; *X. laevis*, group 1; *ceacam325*-*ceacam342; X. laevis*, group 2: *ceacam376*-*ceacam390*. Nucleotide sequences from the N domain exons can be used as gene identifier (Additional files 2 and 3). Gene Nomenclature Guidelines recommended by Xenopus Gene Nomenclature Committee (2013) was followed (http://www.xenbase.org/gene/static/geneNomenclature.jsp).

### Sequence motif identification and 3D modeling

The presence of ITAM, ITAM-like and ITIM/ITSM motifs were confirmed using the amino acid sequence pattern search program ELM (http://elm.eu.org/). Transmembrane regions, glycosylphosphatidylinositol (GPI) signal domains and leader peptide sequences were identified using the TMHMM (http://www.cbs.dtu.dk/services/TMHMM-2.0/), the big-PI predictor (http://mendel.imp.ac.at/sat/gpi/gpi_server.html), GPI-SOM (http://gpi.unibe.ch/) and the SignalP 4.1 programs (http://www.cbs.dtu.dk/services/SignalP/), respectively [40]. For three-dimensional modeling the geno3D software was used (https://geno3d-prabi.ibcp.fr/cgi-bin/geno3d_automat.pl?page=/GENOHLP/genohlp_help2.html). Images were constructed with the Swiss-PdbViewer software 4.1.

### Phylogenetic analyses and determination positive selection

Phylogenetic analyses based on amino acid sequences were performed with the MEGA6 package [41]. The applied Maximum Likelihood method is based on the JTT matrix-based model [42]. The trees with the highest log likelihood are depicted. The percentage of trees in which the protein sequences clustered together is shown next to the branches. Initial tree(s) for the heuristic search were obtained automatically by applying Neighbor-Join and BioNJ algorithms to a matrix of pairwise distances estimated using a JTT model, and then selecting the topology with superior log likelihood value. All positions containing gaps and missing data were eliminated. In order to determine the selective pressure on the maintenance of the nucleotide sequences, the number of nonsynonymous nucleotide substitution per nonsynonymous site (dN) and the number of synonymous substitutions per synonymous site (dS) were determined for N domain exons. The dN/dS ratios as well as the cumulative synonymous and nonsynonymous substitutions along coding regions of N domain exons from paralogous and orthologous genes were calculated after manual editing of sequence gaps or insertions guided by the amino acid sequences using the SNAP program (Synonymous Nonsynonymous Analysis Program; http://www.hiv.lanl.gov/content/sequence/SNAP/SNAP.html). The program *PipMaker* (http://bio.cse.psu.edu/) was used to identify conserved contiguous stretches of nucleotides between gene pairs and to calculate the degree of identity which is summarized as a ‘percent identity plot’ [43]. Multiple amino acid and nucleotide sequence alignments were performed with ClustalW programs (http://npsa-pbil.ibcp.fr/cgi-bin/npsa_automat.pl?page=/NPSA/npsa_clustalw.html).

## Additional files

Additional file 1: ITIM and ITAM/ITAM-like sequence motifs in the cytoplasmic domains of *Xenopus* Ceacam proteins. The amino acid sequences encoded by cytoplasmic domain exons of putative inhibitory (A) or activating *X. tropicalis* and *X. laevis* Ceacams (B) were aligned. Intra- and inter-group 1 and group 2 Ceacam member alignments are shown. The amino acids in one letter code are colored according to their relatedness: red, identical; green, highly similar properties; blue, similar properties. Gaps indicate exon borders, dashes missing amino acids. The names of the cytoplasmic domain-encoding exons and the intron types (0, xxx-intron-xxx; 1, x-intron-xx; 2, xx-intron-x; xxx = codon) are indicated above and below the aligned sequences. ITIM (I/L/V/SxYxxL/V), ITSM (TxYxxV/I), ITAM (D/ExxYxxL/Ix_6-8_YxxL/I) and endocytic or ITAM-like motifs (YxxL/M/V/I/F) are marked by red, orange, green and blue boxes, respectively. Note the characteristic split of the YxxL motif in the ITAM and ITAM-like motifs by phase 0 introns. GRB2-like Src homology 2 (SH2) domains-binding motifs (YxNx) are boxed with magenta lines. Note their conservation in most ITAM/ITAM-like motif-containing Ceacams at presumably non-homologous positions in group 1 and group 2. The small letter x represents any amino acid, and slashes separate alternative amino acids that may occupy a given position. An additional motif (TEHKS) highly conserved in mammals and shown to bind beta-catenin is shaded gray [44]. Note the presence of two types of dissimilar cytoplasmic sequences within (for ITAM-like motifs) and between Ceacam groups (ITIM and ITAM-like) differing in part also in the number and phasing of the encoding exons. Cyt, cytoplasmic domain exon; ITAM, immunoreceptor tyrosine-based activation motif; ITIM, immunoreceptor tyrosine-based inhibition motif; ITSM, immunoreceptor tyrosine-based switch motif; Mdo, *Monodelphis domestica* (gray short-tailed opossum); Xla, *X. laevis*; Xtr*, X. tropicalis*. (DOCX 95 kb)
Additional file 2:
*Xenopus tropicalis ceacam* N exon nucleotide sequences. *Xenopus tropicalis ceacam* N exon nucleotide sequences (Xenbase tropicalis 9.0 Chr07 unless otherwise indicated, e.g. Ensembl Xenopus JGI 4.2). EST, expressed sequence tag; N, N or Ig variable-like domain exon; P, pseudogene (stop codon in N exon). (DOCX 23 kb)
Additional file 3:
*Xenopus laevis ceacam* N exon nucleotide sequences. *Xenopus laevis ceacam* N exon nucleotide sequences (Xenbase laevis 9.1 unless otherwise indicated). EST, expressed sequence tag; N, N or Ig variable-like domain exon; P, pseudogene (stop codon in N exon). (DOCX 22 kb)
Additional file 4: Modeling of the three-dimensional structure of Ceacam301 and Ceacam350 IgV-like domains. Ceacam301 and Ceacam350 mature IgV-like domains were modeled using the geno3D software. The equivalent surface corresponding to the CC’C”FG face is shown as ribbons. The amino acids which flank the CC’C” and FG β-strands are indicated in three-letter code. Amino acids which belong to the CC’C”FG face of the sequence alignment are indicated in yellow. Human and murine CEACAM1 IgV-like domain sequences were used as template for modeling of Ceacam301 and Ceacam350 IgV-like domains, respectively. Note the overall similarity of the putative ligand-binding region shown at the left side despite the fact that the regions corresponding to the C” β-strand in CEACAM1 were not classified as β-strands in Ceacam301 and Ceacam350. (PPTX 190 kb)
Additional file 5: Sequence comparison of leader and N exon genomic regions from *Xenopus* paired receptor *ceacam* genes. Nucleotide sequences comprising N domain exons and flanking regions from presumed paired receptor *ceacam* genes (group 1 and group 2) as well as from the homeologous group 2 inhibitory receptor genes from *X. laevis* were pairwise aligned. Identical nucleotide positions are shown in red. Splice acceptor and donor consensus sequences are marked in yellow and blue, respectively, translational start codons in green. Note extended stretches of nucleotide sequences with high conservation restricted to N exon regions including splice sites but extending barely in to the introns.Xla, *X. laevis*; Xtr*, X. tropicalis*. (DOCX 83 kb)

## Abbreviations

ceacam: Carcinoembryonic antigen-related cell adhesion molecule
dN: Rate of non-synonymous substitutions
dS: Rate of synonymous substitutions
GPI: Glycosylphosphatidylinositol
Ig: Immunoglobulin
IgC: Ig constant
IgV: Ig variable
ITAM: Immunoreceptor tyrosine-based activation motif
ITIM: Immunoreceptor tyrosine-based inhibition motif
ITSM: Immunoreceptor tyrosine-based switch motif
MHC: Major histocompatibility antigens
NK: Natural killer cell
Opa: Opacity-associated
UspA1: Ubiquitous surface protein A1.
