# Supplementary material for: Coevolution of paired receptors in Xenopus carcinoembryonic antigen-related cell adhesion molecule families suggests appropriation as pathogen receptors
Source: BMC Genomics. 2016 Nov 16;17:928. doi: 10.1186/s12864-016-3279-9 (PMC5112662; doi:10.1186/s12864-016-3279-9)
Supplement: Additional file 1: — ITIM and ITAM/ITAM-like sequence motifs in the cytoplasmic domains of Xenopus Ceacam proteins. The amino acid sequences encoded by cytoplasmic domain exons of putative inhibitory (A) or activating X. tropicalis and X. laevis Ceacams (B) were aligned. Intra- and inter-group 1 and group 2 Ceacam member alignments are shown. The amino acids in one letter code are colored according to their relatedness: red, identical; green, highly similar properties; blue, similar properties. Gaps indicate exon borders, dashes missing amino acids. The names of the cytoplasmic domain-encoding exons and the intron types (0, xxx-intron-xxx; 1, x-intron-xx; 2, xx-intron-x; xxx = codon) are indicated above and below the aligned sequences. ITIM (I/L/V/SxYxxL/V), ITSM (TxYxxV/I), ITAM (D/ExxYxxL/Ix6-8YxxL/I) and endocytic or ITAM-like motifs (YxxL/M/V/I/F) are marked by red, orange, green and blue boxes, respectively. Note the characteristic split of the YxxL motif in the ITAM and ITAM-like motifs by phase 0 introns. GRB2-like Src homology 2 (SH2) domains-binding motifs (YxNx) are boxed with magenta lines. Note their conservation in most ITAM/ITAM-like motif-containing Ceacams at presumably non-homologous positions in group 1 and group 2. The small letter x represents any amino acid, and slashes separate alternative amino acids that may occupy a given position. An additional motif (TEHKS) highly conserved in mammals and shown to bind beta-catenin is shaded gray [44]. Note the presence of two types of dissimilar cytoplasmic sequences within (for ITAM-like motifs) and between Ceacam groups (ITIM and ITAM-like) differing in part also in the number and phasing of the encoding exons. Cyt, cytoplasmic domain exon; ITAM, immunoreceptor tyrosine-based activation motif; ITIM, immunoreceptor tyrosine-based inhibition motif; ITSM, immunoreceptor tyrosine-based switch motif; Mdo, Monodelphis domestica (gray short-tailed opossum); Xla, X. laevis; Xtr, X. tropicalis. (DOCX 95 kb) [file 12864_2016_3279_MOESM1_ESM.docx]

A

ITIM motifs

Xenopus Ceacam group 1

(1) Cyt1 (2) Cyt2(1) Cyt3 (0) Cyt4

Xtr_Ceacam301 KQRNENSR SAQMPMS AGVADKNKEDE EYVKYADLRFKNANPANKAPVQQAGETEYSTVKK

Xtr_Ceacam302 KHRNRSSG NAQMTMS -GVADKNQENE EYVKYADLRFKNANPGNKAPVQQVAETEYSTVKK

Xla_Ceacam325 KQTNGSSG NPQMNLT -EVPNKKQEDE EYVKYADLRFKNTNPSNKAETQQVPETEYSTVKR

(1) Cyt1 (2) Cyt2(1) Cyt3 (0) Cyt4

Xtr_Ceacam301 -KQRNENSR SAQMPMS AGVADKNKEDE EYVKYADLRFKNANPANKAPVQQAGETEYSTVKK*

Mdo_CEACAM1 ASDHHPTEHKSSAPNHS HLKSDRPPNGT EEVSYASVNFNAQKSSATVQAPTPTETIYSEIKKK

(2) Cyt1 (1) Cyt2 (0) Cyt3

Xenopus Ceacam group 2

(2) Cyt1 (1) Cyt2 (0) Cyt3

Xla_Ceacam389.L SSKKDTKSTEN GHTSAQGIEM DEAALQYSSIDFSAQRGLQRNEHVVPPPDNTVYAEVRHK

Xla_Ceacam389.S SSKKDKKSTEN GHTSAQGIEM DEAALQYSSIDFSAQQGLQSNDHIVPPFDNTVYAEVRHQ

Xtr_Ceacam350 SSKQEKKSTEN GHTSAQGIEM DESTLQYSSIDFSAQRGLQRNEHVVP--DNTVYAEVRRQ

(2) Cyt1 (1) Cyt2 (0) Cyt3

Xtr_Ceacam350 -----SSKQEKKSTEN- GHTSAQGIEM-- DESTLQYSSIDFSAQRGLQRNEHVVPDNTVYAEVRRQ

Mdo_CEACAM1 ASDHHPTEHKSSAPNHS -HLKSDRPPNGT --EEVSYASVNFNAQKSSATVQAPTPTETIYSEIKKK

Xenopus Ceacam group 1/group 2 ITIM similarity

(1) Cyt1 (2)Cyt2 (1) Cyt3 (0) Cyt4

Xtr_Ceacam301 KQRNENSR SAQMPMS AGVADKNKEDE --EYVKYADLRFKNANPANKAPVQQAGETEYSTVKK*

Xtr_Ceacam350 -SSKQEKK-STEN--- -GHTSAQGIEM DESTLQYSSIDFSAQRGLQRNEHVVPDNTVYAEVRRQ

(2) Cyt1 (1) Cyt2 (0) Cyt3

B

ITAM and endocytic ITAM-like motifs

Xenopus Ceacam group 1

(1) Cyt1 (0) Cyt2 (0) Cyt3

Xtr_Ceacam304 TPTPTNPVYENTGTQAP----------------------SHYDRIIPGSK ENMAGNNPQESSYQ ELQFSNKDVYNNLRKTPR

Xtr_Ceacam315 VPTPTIPVYENTGAQAP----------------------NHYDRIITASK EDMAGNNLQESGYQ ELQFSHNDVYNHLRNAPR

Xla_Ceacam328 --APTSPVYENTGAQAP----------------------NTYDRVITGST ETMAGNRPQESSYQ ELQFPSNDVYNHLRRTPR

Xla_Ceacam334 --APTSPVYENTGAQAP----------------------NTYDRIITGSK ETMAGNRPQESSYQ ELQFPSNDVYNHLRRTPR

Xla_Ceacam332 TPTPTNPVYENTGPQAP----------------------NTYDRVILGSK ENMAESKPQESGYQ ELQFPHNNVYNHLRKTPR

Xtr_Ceacam303 TSTPTNPVYENTGAQAP----------------------NTYDRIITGST ENMARNKPQESSYQ ELQFPHTDLYNNFRKTPR

Xla_Ceacam340 TPTPTNPVYENAEAQMSN DTPTRINPAYENAKAQVSNGNNYDHVIISSE GTMAKNRPQESSYQ VLQFPHIDLYSSLRKTPK

(1) Cyt1 (1) Cyt2 (0) Cyt3 (0) Cyt4

(1) Cyt1 (0) Cyt2 (0) Cyt3 (0) Cyt4 (0) Cyt5

Xla_Ceacam342.L KKQPANETSKIDF QPSGVSNPYM NWHRSTTTVAQQV N-PGVQDEDNYE ELQFQDNIEYDKISP*

Xla_Ceacam342.S -QQAEYITPIKEF PQSGVQNPYM NGPRIATTVQQPV NSPGVAQEEPYT TLECMDNNEYDMIKPSLSFR NVAL

(1) Cyt1 (0) Cyt2 (0) Cyt3 (0) Cyt4 (0) Cyt5 (0)Cyt6

Xenopus Ceacam group 2

(1) Cyt1 (0) Cyt2 (0) Cyt3

Xtr_Ceacam369 --GQSGDRGEDPSQIYYNIPNTAA AHPAIEEGPYM GLQYPSQDTYSELKP*

Xtr_Ceacam371 ---QSGDRGEDPSQIYYNIPNTAA AHPAIEEGPYM GLQYPSQDTYSELKP*

Xtr_Ceacam351 --GQSGDRGEDPSQIYYNIPNTAA AHPTIEEGPYM GLQYPSQDTYSELKR*

Xtr_Ceacam368 --GQSGDRGEDPSRIYDNIPNTAV AHPAKEEGPYM GLQRPSQDTYSELKL*

Xtr_Ceacam375 --GQSGDRGEDPSRIYDNIPNTAA AHPAKEEGPYM GLQRPSQDTYSELKL*

Xla_Ceacam387 ---QSGDIGEDPSRVYENIPNTAK AHPAKEEGPYM GLQYPSQDTYSELKK*

Xtr_Ceacam366 --GQSADRGMDPSQIYDNIPNTAG VQFAKKESEYT GLQHPTQNTYSEMKR*

Xtr_Ceacam359 ---QPTE-GQDSYRIYYNVYAATM AQPAKEELPYM GLEYPTQDTYSELTH*

Xtr_Ceacam364 ---QSSG-RQDSSKIYNNAGNSAM NQSVREESAYM GLLELPQNVYTELKQ*

Xtr_Ceacam361 QTTRQINSRHEQTAPYQNVIEGT SQQNPPVESTYT GLQHQPENAYCNLKMGWRLNR*

Xtr_Ceacam362 ----QFNNKQDSSATYDSVIVGGT GQNISVESPYT GLQHGPDHTYCDLKVWWKRNEKP

(1) Cyt1 (0) Cyt2 (0) Cyt3

Xla_Ceacam379 KYVSIQTTGLVNSGHDQTPPYQNVINGPT QQNPQVE----------SSQYQ GLQHRPENIYFDLKMGGK*

Xla_Ceacam378 KYLSVLKTVKVNSKHDQPTQNPNVIDGTT GQNTVEEPSYP EYSTVDSGYM GLQDRSYHIYNDLDMKGARHG

Xtr_Ceacam360 KYVSVQTTGQAN-RHDGTAHNHN---GTR GQNTTEGPSYP EYSTVDSGYT GLQDRPNQTYSDLNIGGTRHG

(1) Cyt1 (0) Cyt2 (0) Cyt3 (0) Cyt4

Xenopus Ceacam group 1/group 2 ITAM similarity

(1) Cyt1 (0) Cyt2 (0) Cyt3

Xtr_Ceacam303 TSTPTNPVYENTGAQAPNTYDRIITGST ENMARNKPQESSYQ ELQFPHTDLYNNFRKTPR

Xla_Ceacam332 TPTPTNPVYENTGPQAPNTYDRVILGSK ENMAESKPQESGYQ ELQFPHNNVYNHLRKTPR

Xtr_Ceacam304 TPTPTNPVYENTGTQAPSHYDRIIPGSK ENMAGNNPQESSYQ ELQFSNKDVYNNLRKTPR

Xtr_Ceacam315 VPTPTIPVYENTGAQAPNHYDRIITASK EDMAGNNLQESGYQ ELQFSHNDVYNHLRNAPR

Xtr_Ceacam351 ------GQSGDRGEDPSQIYYNIPNTAA ---AHPTIEEGPYM GLQYPSQDTYSELKR*

Xtr_Ceacam369 ------GQSGDRGEDPSQIYYNIPNTAA ---AHPAIEEGPYM GLQYPSQDTYSELKP*

Xtr_Ceacam368 ------GQSGDRGEDPSRIYDNIPNTAV ---AHPAKEEGPYM GLQRPSQDTYSELKL*

Xtr_Ceacam375 ------GQSGDRGEDPSRIYDNIPNTAA ---AHPAKEEGPYM GLQRPSQDTYSELKL*

(1) Cyt1 (0) Cyt2 (0) Cyt3

Xtr_Ceacam304 ---------------- TPTPTNPVYENTGTQAPSHYDRIIPGSK ---ENMAGNNPQ-ESSYQ ELQFSNKDVYNNLR--KTPR-

Mdo_CEACAM110 RGKKDLPRGKQSAPRY --GEDTTVYENT--VHLKGLALPAQ--- GLDSSSTSPEISSESPYQ MLDITRVDVYEKITPWKNPQV

(2) Cyt1 (1) Cyt2 (0) Cyt3 (0) Cyt4
