# Supplementary material for: Coevolution of paired receptors in Xenopus carcinoembryonic antigen-related cell adhesion molecule families suggests appropriation as pathogen receptors
Source: BMC Genomics. 2016 Nov 16;17:928. doi: 10.1186/s12864-016-3279-9 (PMC5112662; doi:10.1186/s12864-016-3279-9)
Supplement: Additional file 2: — Xenopus tropicalis ceacam N exon nucleotide sequences. Xenopus tropicalis ceacam N exon nucleotide sequences (Xenbase tropicalis 9.0 Chr07 unless otherwise indicated, e.g. Ensembl Xenopus JGI 4.2). EST, expressed sequence tag; N, N or Ig variable-like domain exon; P, pseudogene (stop codon in N exon). (DOCX 23 kb) [file 12864_2016_3279_MOESM2_ESM.docx]

***Xenopus tropicalis* *ceacam* N exon nucleotide sequences (Xenbase tropicalis 9.0 Chr07 unless otherwise indicated, e.g. Ensembl Xenopus JGI 4.2)**

EST, expressed sequence tag; N, N or Ig variable-like domain exon; P, pseudogene (stop codon in N exon)

**Group 1**

>Xtr_ceacam301N

GTACTGTAAGATCTGTAGAGAATGTAAATGGGACTGTGGGCAAATCTGTGTATCTCACAGTGAAGTTGGATCTCCCGGCACAGAGGCAGGTAACATGGAAGGTTAATTCCAGCACGCAGATTGTAACTGCAGTAACTGGAGGCTCTCCCGTTTATTTTGGAAGCTACGGAGACAGATGCACCCTGTATGAGAACACAACTCTCCGGCTGGACAATCTCACCCCCACAGATACAGGGGAATATACACTCACTGTAGCCAACGTGAGCACTGGATCAACACAATCAGGATCAGTTTATCTCACAGTTTACA

>Xtr_ceacam302N not in Xenbase tropicalis 9.0, cytoplasmic domain exons on scaffold_3747; EST EG656078

GGTCTGTATCCTTACAGAATGTCTTTGAAGGGATAGTGGGTGGATCTGTGAATCTCACTGTGAAGTTGAATCTGCCAGCAAATAGGTTGGTACAATGGAAGTTTAAAACCAGCACTCTAATTGTAACTGGATTACCTGGCGGCTCCCCTTTATATACTGATGACTACAAAGGCAGATGCCACCTGTATGAGAACTCAACTCTCCGGCTGGACAATCTCACTCCCGCAGATGAGGGGAACTATACACTCTCTGTAACCAACCAAGGCAATGGAGATACAGAAACAGGATCAGTTATGCTCTTGATTTACA

>Xtr_ceacam303N

GTACTGTAAGATCTGTAGAGAATGTAACTGGGACTGTGGGCAAATCTGTGTATCTCACAGTGAAGTTGGATCTCCCGGCACAGAGGCAGGTAACATGGAAGGTTAATTCCAGCACGCAGATTGTAACTGCAGTAACTGGAGGCTCTCCCGTTTATTTTGGAGACTACGGAGGCAGATGCACCCTGTATGAGAACACAACTCTCCGGCTGGACAATCTCACTCCCACAGATACAGGGGAATATACACTCACTGTAGCCAACACGGGCACTGGAGCAACACAATCAGGATCAGTTTATCTCACAGTTAACA

>Xtr_ceacam304N

GGTCTGTATGCTGCCTACAGAGTGTATCAGGGATTGTGGGTGGATCTGTGACTCTCACAGCGATTCTAAATGTACCTGCACAGCCGGTGGTAACATGGAGGTTTGGTACCGATCAGATTGCATATGCAGCACTGGGTAATTCTGCTGTTTATAATAATAGCTGCACAGACAGATGCACCCTGTATGGGAACGCTAGTCTCCGGCTGGGCAGTCTCACTCCCGCAGATACAGGGGAATATTCACTCACTGTACTTAATGTTCCGGCTGGAACACAACAAACAGAACTGTTTCATCTCTCAGTTTACA

>Xtr_ceacam305P_N

GTGCTGTAAGATCTGTAGAGAATGTAACTGGGACTGAGGGCAAATCTGTGTATCTCACAGTGAAGTTGGATCTCCCGGCACAGTGGCAGGTAACATGGAGGGTTAATTCCAGCAAGCAGATTGTAATTGCATTACCTGGAGGCTCTCCCGTTTATTCTGCAGACTACGGAGACAGATGCACCCTGTATGAGAACGCTAGTCTCCGGCTGGACAATCTCACTCCCACAGATACAGGGAATATTCACTCACTGTAACCAACCTGGGCGCTGTAGGAACACCATCAGTGACAGGATCAGTTTATCTCACAGTTTATG

>Xtr_ceacam306P_N

GGGTCGCCCCATTTAGATATTGCACCTATGACTGAAGGTGGATCTGTATATCTCAGTGTGGGGACTAATCTGCCTAAAAACCGCCAGTTACAATGGAGGTTTAATACCAGCACTATGATTGCATATGGAATTACTGGAGGCTCTCCCATTTATTTTGGACATGCTCCCTGTACGAAAACACAACTCTCCGGCTGGACAATCTCACCGCCGCAGATACGGGGGAAATATTCACTCACTGTAACCAACCTGGGCACTGGAACAGTACAAACTGGATCAGCGTATCTCACAGTTAACA

>Xtr_ceacam307N not in Xenbase tropicalis 9.0; Ensembl scaffold GL186323.1

GTGCTGAAAGATCTATAGATAATGTAGCTGGGACTGAGGGCAAATCTGTGTATCTCACAGTGAGGTTGGATCTCCCGGCACAGCGGCAGGTGACATGGAGGGTTAATTCCAGCTCCCAGATTGTGACTGCAGTTACTGGAGGCTCTCCCGTTTATGCTGCAGACTACAGAGACAGATGCCACCTGTATGGGAACGCAACTCTCCAGCTGGACAATCTCACTCCCGCAGATACAGGGGAATATACACTCTCTGTAGCCAACGTGAGCACTGGAGCAACAGAATCAGGATCAGTTTATCTCACAGTTTACA

>Xtr_ceacam308N

GTGCTGTAAGTTCTGTAGAGAATGTAACTGGGACTGAGGGCAAATCTGTGTATCTCACAGTGAAGTTGGATCTCCCGGCACAACGGCAGGTTACATGGAAGGTTAATTCCAGCATTCAGATTGTAACTGCATTACCTGGAGTCTCTCCTGTTTATACTGACAGTTACAGAGACAGATGCTCCCTGTATGGGAACACAACTCTCCGGCTGGACAATCTCACTCTCACAGATACAGGGGAATATTCACTCTCTGTAACCAACCTGGAAACTGGAGCACCACCAGTGACAGGATCAGTTTATCTCACAGTTTACA

>Xtr_ceacam309N

GGGCTGTGGTTTCTGTAGAGAATGTGCAGGGCATTGAAGGCCAATCTGTGACTCTCAGTGTAAATCTGACACTGTTTGAAAATCAGATGATAACATGGTATTTTAATAAATACAGTTTGGTCGCTTCAGAAACAACGAACAACGCGCCAAGTTTTTTTGGGGGGTATGAAGGGAGATGCACCCTGTTTGAGAATGCAACCCTCCAGCTGGACAATCTGACACCTGCAGACCAGGGCAATTACACACTCACTGTACTGAACTTGGATACAGGATTGTCTGTGTCAGGATTGGTCTGTCTCACAGTACAGA

>Xtr_ceacam310P_N

GATCTGTATCTTGTTTACAGGTAGCGGGGACTGAGGGCAAATCTTTTTATCTTTCCATAAAGTTAAATCTCCCTGCACAGCGGTTGGTACAATGGGGCTTTGGTACAAACATTCACATTGTAACTGCATTCCCGGATACAACTCCCCAGTATTTTGGTAGCTACAGAGGCAGATGCATCCTGTATGAGAACACAACTCTCCAGCTGGACAATCTCACTCCCGCAGATACAGGGGAATATACACTCACTGTAACCAACATGGACACTGGAGCAATACAATCAGGATCAGTTTCTCACAGTTTACA

>Xtr_ceacam311N

GGGCTGTGGTTTCTATAGAGAATGTGCAGGGCATTGAAGGAGAATCCGTGACTCTTAGTATAAACCTGAATGTGACTGTATTTATATTGATAGAATGGAGCTTTGATTCACACAGTATGATAGCAATGCTAACGGGAAACAGTCCAATATATTTTGGGGAGTATGAGGGGAGATGCACCCTGTTTGAGAATGCAACTCTCCAGCTGGACAATCTGACACCTGCAGACCAGGGCAATTACACACTCACTGTACTGGATGGAGAGACCGGAATGCCAGTGTCTGGATCAGTCTGTCTCTCAGTACAGG

>Xtr_ceacam312N

GATCTGTATCTTTTTTAGTGGATGTGCAGGGATACAAGGGTACTTCTGTGAATCTCACTGTGAAGCTGCCCCACCCTACAAATAGGTTGATACAATGGACATTTAATTCCAGCAGTCCAATTGTGACTGTGATGCCAGATTTGCCTTCCTCATATACTGACAGTTACAGAGGCAGATGCACCCTGTATGAGAACACAACTCTCCAGCTGGACAATCTCACTCCCGCAGATACAGGGGAATATTCACTCTTTGTAACCAACCTGGACACTTCAGTGCAAGAAATAGGGTCAGTTCATCTCACAGTTTACA

>Xtr_ceacam313N1

GGTCAATATCTTTTTTCATGTTGTTGCATGGAATTGAGGGCAGAGCAGTGTATTTCCCTATATATATGACACTCCCTAAAAATCATCGAAAACAATGGTGGTTCGATACCAGCACTCTGATTGTAACTGATTTTTCAAATGGCATTACTTCTTATACTGAGGAATACAAAGACAGGTGCTCAATATACATTAACTCAACTCTACGGATGGACAAGCTCACTTTAGCCGACCAGGGAATGTACAGTCTGAATGTAACCAACTTGGATAATGGACATTCCCAATCAGAATCACTTTATCTCATGGTTTACA

>Xtr_ceacam313N2

AATTTGAACCATCTATAGAGAATATAACTGGGATTGAAGGTGGGTCTGTGCATCTCAGTGGGAATCTGCCTTTCCCTATACGCTGGAATGTACAATGGAACTTTAATAGCAGCACCAAGATTTTAACTGCAAGACCAAACAGCCCTCCTGATTATTCTGAAAAATACAGAGATAGGTGCATTATGTATGAGAATGCAACCCTCAGCCTGGATAAACTCACTCCCGCAGATCAGGGGGAATATGAGTTTGCTGTAATCGATGTGGAAAATGAAAAAGTAAAGACAGCATTTATTTATCTCACAGTTTACG

>Xtr_ceacam313N3

GAATATATAGAGAGAATATAACTGGGAATGAAGGTGAGTCTGTGTATCTCAGTGGGAATCGGCCTCTCCCTATATGCTGGAAGGTACAATGGAACTTTAATAGAAGCACTGAGATTTTAACTGCAAGATCTAATGGCTCAACTAATTATTCTAAAAAATACAGAGATAGGTGCATTATGTATGAGAATGCAACCCTCAGGCTGGATAAACTCACTCCCGCAGATCAGGGGGAATATGAACTCACTGTGATTGAAGAGGACAATGGAAAAGTCCAGACAGCATTTCTTTATCTTACAGTTTACA

>Xtr_ceacam314N

GGGCTGTGGTTTCTATGGATAATGTGCAGGTTTTTGAAGGAGAATCTGTGACTCTCAGCGTAAATCTGAACATGTCTGAATATCAGATGATAACATGGAAGTTTGATACATACACCCTAGTTGCAATAAAAACAGTAAACAACACTCCAACTTGTTTTCCCGAGTATGAGGGGAGATGCACCCTGTTTGCGAATGCAACTCTCCTGCTGGACAATCTGACACCTGCAGAGGAAGGAAATTATACTCTGACTGTAATGGATGTGGAGACCGGAGCGTCTTTGTCAGGATCAATCTTTCTCTCAGTACAGG

>Xtr_ceacam315N

GCCCTGTATGTTGTTTGCTGGAGTCAGGGGAGTTGGGAAAATCTGTATATCTCACTGTGAAGCTAAATCTGCCCGCACAGCGTGAAGTACAATGGAGATTTGGTACCAACACTCTGATAGTATCTGCCCAGCCGGGTATCCCTCCCATCTATTATGGTGCCTACAGAGGCAGATGCACCCTATATGAGAACACAACTCTCCAGCTGGACAGTCTCACTGATGCAGATACAGGGCAATACACACTCTCTGTAATCAATGTGGACACTGGAGCACAACAACAGGGGTCTGTTAATCTCACTGTTCACA

>Xtr_ceacam316N

GATCTGTATCTTGTTTACAGGTAGAGGGGATTGTGGGCGAATCTGTTAATCTTTCCATAAAGTTAAATCTCCCTGCACAGCTGTTGGTACAATGGGGCTTTGGTACAAACATTCACATTGTAACTGCATTCCCGGATACAACTCCCCAGTATTTTGGTGCCTACAGAGGCAGATGCACCCTGTATGGGAACACAAATCTCCGGCTGGACAATCTCACTCCTATAGATACAGGGGAATATACACTCACTGTAACCAACATGGACACTGGAGCAATACAATCAGGATCAGTTTATCTCACAGTTTACA

>Xtr_ceacam317P_N

GGTCTGTATGCTGCCTACAGAATGTATCAGGGATTGTGGGCGAATCTAAGACTCTCACAGCGACGCTAAATCTGCCTGCACAGCCAGCGGTAACATGGAGGTTTGGTACCGATCAGATTGCATATGCAGCACTGAGTAATTCTGCTGTGTATAATAATAGCTGCATAGGCAGATGCACCCTGTATGGGAACGCTAGTCTCCGGCTGGACAATCTCACTCCCTCAGATACAGGGGAATATTCACTCACTGTAACCAACTTTCAGACTGGAACATAACAAACAGAACTGTTTCATCTCTCAGTTTACA

>Xtr_ceacam317N

GTGCTGTAAGATCTGTAGAGAATGTAACTGGGACTGAGGGCAAATCTGTGTATCTCACAGTGAAGTTGGATCTCCCAGCACAGCAGCAGGTAACATGGAGGGTTAATTCCAGCACTCAGATTGTGACTGTGTATCCTGGCGGCTCTCCCATATATACTGATAGTTACAGAGACAGATGCACCCTGTATGGGAACACAACTCTCCGGCTGGACAATCTCACTCCCACAGATACAGGGGAATATTCACTCTCTGTAACCAACCCGAACTCTGGAGCAACACAATCTGGATCAGTTTATCTCACAGTTTACA

>Xtr_ceacam318N1 not in Xenbase tropicalis 9.0; EST EL661387, EG655648

GGTCTGTATGCTGCCTACAGAATGTATCAGGGATTGTGGGTGGATCTGTGACTCTCACATCGACGCTAAATCTGCCTGCACAGTCTACGGTAACATGGATGTTCGGTACCGATCGGATTGCATATGCAGCACTGAGTGATCCTGCTATTTATGATAATAGCTGCATAGGCAGATGCACCCTGTATGGGAACACAACTCTCCGGCTGGGCAGTCTCACTCCCGCAGATACAGGGGAATATACACTCTCTGTATACAACGTTCTGTCGGGAACGCAACAAACAGAACTGTTTCATCTCTCAGTTTACA

>Xtr_ceacam318N2 not in Xenbase tropicalis 9.0; EST EL661387, EG655648

GTGCTGTAAGATCTGTAGAAACTGTAGCTGGGACTGTGGGCAAATCTGTGTATCTCTCTGTGAAGTTGGATCTCCCGGCACAGCGGCAGGTAACATGGAGGGTTAATTCCAGCACTCAGATTGTGAATGCGTATCCTGGCGGCTCTATCATATATTCTGATGCCTACAGAGACAGATGCACCCTGTATGAGAACACAACTCTCCGGCTGGACAATCTCACTCCCGCAGATACAGGGAACTATACGCTCACTGTAACCAACTCGACCCCTGTAGTACCAGTAACAGAATCAGGATCAGTTTATCTCACAGTTTACA

>Xtr_ceacam319N not in Xenbase tropicalis 9.0; Ensembl scaffold GL189012.1

GTGCTGTAAGTTCTGTAGAGAATGTAGTTGGGACTGTGGGCAAATCTGTGTATCTCACAGTGAAGTTGGATCTCCCGGCACAGCGGCAGGTAACATGGAAGTTTAATTCCAGCATTCAGATTGTAATTGCATTACCTGGAGGCTCTCCCACTTATAATGGAGACTACAGAGACAGATGCACCCTGTATGGGAACACAACTCTCCGGCTGGACAGTCTCACTCCCACAGATACAGGGGAATATTCACTCACTGTAGCCAACATGGACACTGGAGCACCACAATCAGGATCAGTTTATCTCACAGTTTACA

>Xtr_ceacam320N Xenbase tropicalis 9.0 scaffold_1090

GTGCTGTAAGATCTGTAGAGAATGTAAATGGGACTGTGGGCAAATCTGTGTATCTCACAGTGAAGTTGGATCTCCCGGCACAGAGGCAGGTAACATGGAAGGTTAATTCCAGCTCCCAGATTGTAACTGCATTACCTGGAGGCTCTCCCGCTTATAATGGAGACTACAGAGACAGATGCACCCTGTATGGGAACACAACTCTCCGGCTGGACAATCTCACTCCCACAGATACAGGGGAATATTCACTCACTGTAACCAACCTGGACACTGTAGGACCACTATTGACAGGATCAGTTTATCTCACAGTTTACG

>Xtr_ceacam321N not in Xenbase tropicalis 9.0; EST CX983103 GCCCTGTATGTTGTTTGCTGGAGTCAGGGGAGTTGGGAAAATCTGTAAATCTCACCGTGAAGCTAAATCTGCCCGCACAGCGTGAAGTACAATGGAAGTTTAGTACCAACACCGTGATAGTATCTGCCCCGCCGGGTATCCCTCCCACCTATTATGGTGCCTACAGAGGCAGATGCACCCTGTATGAGAACACAACTCTCCAGCTGGACAGTCTCACTGACGCAGATACAGGGCAATACACACTCTCTGTAATCAATGTGGACACTGGAGAACAACAACAGGGGTCTGTTAATCTCACTGTTCATA

**Group 2**

>Xtr_ceacam350N

TTTCCCTCAGTGCTTGGATGGATGGAGCCCATGGAATTGGGGTTCAGCTGATCCCTCAGTATCCGGTGGTCAGTCAGTCTGTTACTCTGAGTGTCACTGGGGTCACTGGCACCATACGGCAGTTCGACTGGTTTAAAGGTTCAAGTGCAGATACCAAAAACCAAATATTCAGTGTTATTCCACCTTTAAACACAGTGACAGAAGGGCCTCAGTATTTCCCTCGTGCCAATTGGTTCCCAAATGGCTCATTGCAGATCTCAGGCCTTGTTCCTACAGACCAGGGGAATTACACAGTGCTGATACAGACTGCTGAGAGTGTAACACAAGCTACAGTTTCCCTGCCAGTTTATG

>Xtr_ceacam351N

TTTCCCTCAGTGCTTGGATGGATGGAGCCCATGGAATTGGGGATCAGCTGATCCCTCAGTATCCGGTGGTTAGTCAGTCTGTTACCCTGAGTGTCACTGGGGTCACTGGCACAGTAATCGCATTCGCATGGTATAAAGGTTCAAGTGCAGATACCAATAACCAAATATTCATTGTTATTCCATCTTTAAACTCAGTGACAGAAGGGCGTCAGTATTTCCCTCGTGCCAATTGGTTCCCAAATGGCTCATTGCAGATCTCAGGCCTTGTTCCTACAGACCAGGGGAATTACACAGTGCTGATAGAGACTGCTGAGAGTAGAGCACAACATACAGTTTCCCTGCCAGTTTATG

>Xtr_CEACA353P_N stop in transmembrane domain exon

TTTCACTCAATGCTTGGATGGATGGAGCCCATGGAATTGGGGGTCAGCTGATCCCTCAATATCCAGTGCTTAATCAGTCTGTTACCCTGAGCGTCACTGGGGTCACTGGCACCATACGGAAGTTCACATGGTACAAAGGTTCAAGTACACATGATAGCCAAATATTCAGAGTTATTTTATCTCTAGACTCAGTGACAGAAGGGCCTCAGTATTTCCCCCATGTCAGTCAGTTCCCAAATGGCTCATTGCAGATCTCGGGCCTTGTTCCTACAGACCAGGGGACTTACAGAGTGCTGATACAGTCTGGGAGTATAACACATTTATTACAAGTTTTTGTTACTGTTTATG

>Xtr_ceacam354N

TTTCCCTCAGTGCTTGGATGGATGGAGCCCATGGAATTTGGGTTCAGCTGATCCCTCAGTATCCGGTGCTTAATCAGTCTGTTACCCTGAGCATCACTGGGGTCACTGGCACAATACGGCAGTTCGACTGGTATAAAGGTTCAAGTGTAGATACCAATACCCAAATATTCAGTGTTATTCCATCTGCAAACTCAGTGACAGAAGGGCCTCAGTATTTCCCTCGTGCCAATTGGCTCCCAAATGGCTCATTGCAGATCTCAGGCCTTGTTCCTACAGACCAGGGGAATTACACAGTGCGGATACAGACTGCTGAGAGTACAGTACAAGCGACAGTTTCCCTGCCAGTTTATG

>Xtr_ceacam355N

CTCTCCTCAGCTTTCTGATGGACACCACAAGAGCTATCACCATTGAAGTGATCCCTAAACTTCCAACTCCCAATTCAGATGTTACTCTCAAAGTTAAAGGAGTCTCTGGGATAATAAGAAGCTTCAGCTGGTACAATGGATCGAACCCAAGTGCTTCAAATCAAATCTTAAACTATATCCCATCTCTTCAACCACCACAGACCAAAGGACATATGTACTTCCCCCAAGCCGAAGGACTGCCAAATGGCTCACTTCTGATCAAAGACTTTGTGAAGAAATTTGAGGGTGTCTACACAGTGCAGATACAAGCAGACAGCCCACTGCAAGCATCAGTATCAGTAACCATGAGCG

>Xtr_ceacam356N

TTTCCCTCAGTGCTTGGATGGATGGAGCCCATGGAATTGGGGTTCAGCTGATCCCTCAGTACCCGGTGGTTAGTCAGTCTGTTACCCTGAGCGTCACTGGGGTCACTGGCACCATACGGCAGTTCAGCTGGTATAAAGGTTCAAGTGCAGATACCAATAACCAAATATTCAGTGTTATTCCATCTGCAAACTCAGTGACAAAAGGGCCTCAGTATTTCCCTCGTGCCAATTGGCTCCCAAATGGCTCATTGCAGATCTCAGGCCTTGTTCCTACAGACCAGGGGAATTACACAGTGCTGATGTATACTGCTGAGAGTACAACACAAGATACAGTTTCCCTGCCAGTTTATG

>Xtr_ceacam357N Xenbase tropicalis 9.0 scaffold_2548

TTCTCCTCTCTCTATGCATGGTTGCTGCCGGCAGCCTTACCATTAAGTTGAAACCAGACTGTGCATCGGTTGGCAAGAATGTCACTCTCAGTGTCAGTGGAATAAATGGGAACATAACCAGCTTTACCTGGTATCTAGGAGAGCCAAGTGCTTCAAACCAGATAATAAACTATGCAGTAAGCTTTACGCCTCCCAGCGCTCCCGGACCCAAAAATTTTTCAGATGCCATTGGACTTCCAAATGGTTCCTTGCTGATTACAAACCTTAAGACAGAATACAGTAATACCTACACGGTACAGGTACAGGCAGACACACCGGGCCAAGCTTCAGCTGAGCTGACTGTGCAAA

>Xtr_ceacam358N

TTCTCCTCTCTCTGTGCATGGTTGCTGCCAGCAGCCTTACCATTCAGTTGATCCCAGACTGTGCATCGGTCGGCAAGAACGTCACTCTCAGTGTCAGTGGAATAAATGGGAACTTAAATAGCTTTACCTGGTATCTAGGAGAGCCAAGTGCTTCAAACCAGATAATAAACTATATAGTAAGCATTACACCTCCCAGCGCTCCCGGACCCAAAAACTTTTCTGATGCCATTGGACTTCCAAATGGTTCCTTGCTAATTACAAACCTCAAGACAGAATACAGTAATACCTACACGGTACAGGTACAGGCAAGTACACCGGGCCAAGCTTCAGCTGAGCTGACTGTGGAAA

>Xtr_ceacam359N

TTTTCATCAGTCTTTGGATAGAGTCAGGCTATGGAACTGATGTTCAGCTAATCCCCAGCCATCCATTGGTCAATAAGTCTGTTACCTTGAATGTCAGAGGAATCACAGGTACATTACGCTACTTCAGGTGGTATTTAGGGTCAAGGATAGATGCTGCCAACCAAATCTTACACTACAATCCAAATGTTAACCCCCCGCAATTACCAGGTGTTCAGTATTTCCCTAGGGCCCATGGGCTCCCAAATGGCTCCTTACATATCTCAGACCTTGCTCATACAGACCAGGGGATGTACACAGTGATGATACTTACAGGAGGTATGGAAAGATTAACCGTTCATCTGCCTGTTTATG

>Xtr_ceacam360N

CTCTCTTCAGTGTCTGGATAAATCTGATCTATGGAATCAAAATTCAGGCGATTCCTAAATATCCAGTGGTCAATAAGCCTGTCACCCTCAGTGTCAGTGGGGTCAGCGGGGCCATTCGCTCTTTCTCCTGGTATAGTAGTTCAGCCATTTGGAATTCCTCCCTAATTTTATCATATAATCCAACTTCGAACCCTGTGGAGACACAAGGGCCAAAGTACTTCCCTCGAGCCAGCAGCTTCTCAAATGGATCATTAAGGATCTCAAAACTCTTTACTACAGACAGAGGATATTACACAGTGCAAATCCAGGCAGAAAGTTCAACCCAAGAAACTATTAACCTACCTGTATATG

>Xtr_ceacam361N

CTCTCTTCAGTGTCTGGATAAATCTGATCTATGGAATCAGTATTCAGCCGATTCCTGAATATCCAGTGGTCAATCAGCCTGTCACCCTCAGTGTCAGTGGGGTCAGCGGGGCCATTCGCTCTTTCTCCTGGTATAAAGGTCAATTTGTTGATGATTCCTCCCTAATCCTAGCATATAATCCAACTTCTTACCTTGTGGCGAGACGAGGGCCAGAGTACTTCCCTCGAGCCAGTGTCCTCCCAGATGGATCATTAAAGATCTTCAAAATTTATACAGCAGTCAGAAGATATTACACAGTGCAAATCCAggcagaaagtttaacccaagaaaccattaacctgcctgtatatg

>Xtr_ceacam362N

CTCTCTTCAGTGTCTGGATAAATCTGATCTATGGAATCAGTATTCAGGCGATTCCTAAATATCCAGTGGTCAATCAGTCTGTCACCCTCAGTGTCAGTGGGGTCAGCGGGGCCATTCGCTCTTTTTCCTGGTATAAAAGTTCATCCATTACGAATTCCTCCCTAATCTTAACGTATAATTCATCTTCTAACCCTGTGGAGACACAAGGGCCACAGTACTTCTCTCGGGCCAGCGGCCTCTCAGATGGATCATTAAGGATCTCAACCCTTTATACTTCAGACCAGACAAGTTACACTGTGCAGGTTGAGGCAAGCAGTTTAACAAAAGACTCGATTTACCTGCGTGTTTATG

>Xtr_ceacam363N

CTCTCTTTAGTGTCTGGATAAATCTGATCTATGGAATCAGCATTCAGCCGATTCCTGAATATCCAGTGGTCAATCAGCCTCTCACCCTCAGTGTCAGTGGGGTCAGCGGGGCAATTCGCTCTTTTTCCTGGTATAAAAGTTCATCCATTGCGAATTCCTCCCTAATCTTAACGTATAATTCATCTTCTAACCCTGTGGAGACACATGGGCCACAGTACTTCTCTCGGGCCAGCGGCCTCTCAGATGGATCATTAAGGATCTCAACCCTTTATACTTCAGACCAGACAAGTTACACTGTGCAGGTTGAGGCAGGCAGTTTAACAAAAGACTCGATTTACCTGCGTGTTTATG

>Xtr_ceacam364N

TTTCCCTCAGTGTTTGGATGGATGGAGCCCATGGAAGTGGGGTTCAGCTGATCCCTCAGTATCCGGTGGTTAATCAGTCTGTTACCCTGAGTGTCACTGGGGTCAATGGCACAATAGAAAAGTTTACATGGTATAAAGTTTTAAATGCACATGATTATAACCAAATTCTCAGCGTTATTCCATCTCTAAACACAGTGACAAAAGGGCCTCAGTATTTCTCTCGGGCCAGTCAGTTCCCAAATGGCTCATTGCAGATCTCAGGCCTTGTTCCTGCAGACCAGGGGACTTACACAGTGCAGATACAGACTGCTGAGAATATAACATGGTTTCCAGTTCTGCTGCCAGTTTATG

>Xtr_ceacam365N1

TTTCCCTCAGTGTTTGGATGGATGGAGCCCATGGAATAGGGGTTCAGCTGATCCCTCAGTATCCGGTGGTTAATCAGTCTGTTACCCTGAGGGTCACTGGGGTCACTGGCACAATACGGCAGTTCACATGGTATAAAGGTTCAAGTACAGATAGCAATAACCAAATATTCAGTGTTATTCCATCAGCAAACTCAGTGACAAAAGGGCCTCAGTATTTACCTCGTGCCAGTTGGTTCCCAAATGGCTCATTGCAGATCTCAGGCCTTGTTCCTACAGACCAGGGGAATTACACAGTGCAGATACAGACTGAGAATTTAACACAAGTTACAGTTCTGCTGCGAGTTAATG

>Xtr_ceacam365N2

TTTCTCTTTCAGATTGGAAGCATGGAGCCCATGGATTTGGGGTTCAGCTCATCCCTCAGTATCCAGTGGTTAATCAGTCTGTTACCTTGAGAGTCACTGGGGTCGCTGGCACAATACTGCAATTCTGGTGGTACAAGGGGTCAAGTGTAGATACCAATAGCCAAATATTCAGTGTTATACCATTTCCAAACTCAATGACACCAGGGCCTCAGTATTTCCATAGGGCCAGTCAGTTCCCAAATGGCTCATTACAGATCTCAAGCCTTATTCCTACAGACCAAGGGAATTTTGCACTGATGATGCTGACTGCTCAGGGTTTAGCAAAGGTTTACATTTACCTGCCAGTTCATG

>Xtr_ceacam366N

CTCTCTTCAGTGTCTGGATAAATCTGATCTATGGAATCAATATTCAGCTGATTCCTAAAAATCCAGTGGTCAATCAGTCTGTCACCCTCAGAGTCAGTGGGGTCAGCGGGGCCATTCTCTCTTTCTCCTGGTATGGAGGTTTATCAGATGACGACAATATGTTCCTAATATTAACCTACAATTTATCTTCTTACCCTGTGGAGAAACGAGGGCCACTGTACATTCCAGGGGCCAGGGGCCTTCCAGATGGATCATTAAAGATCTCAAAACTTTCTACTGCATATAGAGGAATTTTCACAGTGGGAATACAAGCAGAAAGTTTCCCAACCAAAGGAAGTATTTACCTACCTGTATTTG

>Xtr_ceacam367N

CTCTCTTCAGTGTCTGGATAAATCTGATCTACGGAATCAATATTCAGCTAATTCCTAAATATCCAGTGGCCAATCAGCCTGTCACCCTCAGTGTCAGTGGGGTCAGCGGGGCCATTCTCTCTTTCTCCTGGTATAGAAGTTCAGCCATTATGAATTCCTCCCTAATTTTATCATATAATCCAACTTTGAACCCCGCGGAGACCCAAGGGCCACAGTACTTCCCTCGAGCCAGCAGCTTCTCAAATGGCTCATTACATATAGTAGAACTTTCAACAACAGACAGAGGATATTACACAGTGCAAGTACAGGCAGGAAGTTTAACATCACAGACTATAGACCTGCCTGTATATG

>Xtr_ceacam368N

TTTCCCTCAGTGCTTGGATGGATGGAGCCCATGGAATTGGGGTTCAGCTGATCCCTCAGTACCCGGTGGTTAATCAGTCTGTTACCCTGAGTGTCACTGGGGTCACTGGCACAATACGGCAGTTCAGCTGGTATAAAGGTTCAAGTACAGATACCAATAACAATATATTCAATGTTATTCCATCTGCAAACTCAGTGACACCAGGGGCTCAGTATTTCCTTCGTGCCAATTGGTTCCCAAATGGCTCATTGCAGATCTCAGGCCTTGTTCCTACAGACCAGGGGAATTACACAGTGCTGATAGTGACTGCTGAGAGTGTAACACAAGCTACAGTTTCCCTGCCAGTTTATG

>Xtr_CEACAM369N not in Xenbase tropicalis 9.0; Ensembl scaffold GL173633.1 ENSXETG00000030756

TTTCCCTCAGTGTTTGGATGGATGGAGCCCATGGAATTGGGGTTCAGCTTATCCCTCAGTATCCGGTGGTTAATCAGTCTGTTACCCTGAGCATCACTGGGGTCACTGGCACAGTAAGCGCATTCCTATGGTATAAAGGTTCAAGTGCAGATACCAATAACAATATATTCAGTGTTATTCCATCCGTAAACATAGTGTCAAAAGGGCCTCAGTATTTCCCTCGTGCCAATTGGTTCCCAAATGGCTCATTGCAGATCTCAGGCCTTGTTCCTACAGACCAGGGGAATTACACAGTGCAGATAGTGACACCTGGGAATGCAGCACAACATACAGTTTCCCTGCCAGTTTATG

>Xtr_ceacam370N not in Xenbase tropicalis 9.0; Ensembl scaffold GL173633.1 ENSXETG00000034104

TTTCCCTCAGTGCTTGGATGGATGGAGTCCATGGAATTGGGGTTCAGCTGATCCCTCAGTACCCGGTGGTTAGTCAGTCTGTTACCCTGAGCGTCACTGGGGTCACTGGCGCCATACGGCAGTTCACATGGTATAAAGGTTCAAGTACAGATACCAATAACCAAATATTCAGTGTTATTCCATCTTACAGCTCAGTGACAGAAGGGCCTCAGTATTTCCCTCGTGCCAGTCAGTTCCCAAATGGCTCATTGCAGATCTCAGGCCTTGTTCCTACAGACCAGGGGAATTACACAGTGCTGATACAGACTGCTGGGAGTAGAGCACAACATACAGTTTCCCTGCCAGTTTATG

>Xtr_ceacam371N

TTTCCCTCAGTGTTTGGATGGATGGAGCCCATGGAATTGGGGTTCAGCTGATCCCTCAGTATCCGGTGGTTAGTCAGTCTGTTACCCTGAGCGTCACTGGGGTCACTGGCACAATACGGCAGTTCGACTGGTATAAAGGTTCAAGTACAAATACCAATACCCAAATATTCACTGTTATTCCATCTACAAACTCAGTGACAAAAGGGCCTCAGTATTTCCCTCGTGCCAGTCAGTTCCCAAATGGCTCATTGCAGATATCAGGCCTTTTTCTTACAGACCAGGGGAGTTACACAGTGCAGATACAGACTGAGAGTACAGTACAAGCTACAGTTTCCCTGCCAGTTTATG

>Xtr_ceacam372N

TTTCCCTCAGTGTTTGGATGGATGGAGCCCATGGAATTGGGGTTCAGCTGATCCCTCAGTATCCGGTGGTTAGTCAGTCTGTTACCCTGAGCGTTACTGGGGTCACTGGCACCATACGGAAGTTCACATGGTACAAAGGTTCAAGTACACATGGTAGCCAAATATTCAGAGTTATTTCATCTCTAGACTCAGTGACAGAAGGGCCTCAGTATTTCTCCCATGTCAGTCAGTTCCCAAATGGCTCATTGCAGATCTCAGGCCTTGTTCCTTCAGACCAGGGGACTTACAGAGTGCAGATACAGGCTGGGAGTATAACACATTTATTACAAGTTTTTGTTACTGTTTATA

>Xtr_ceacam375N

TTTCCCTCAGTGCTTGGATGGATGGAGCCCATGGAATTGGGGATCAGCTGATCCCTCAGTACCCGGTGGTTAGTCAGTCTGTTACCCTGAGTGTCACTGGGGTCACTGGCACAATACGGCAGTTCACATGGTATAAAGGTTCAAGTGCAGATACCAATAACCAAATATTCAGTGTTATTCCATCAGCAAACTCAGTGACAGAAGGGCCTCAGTATTTCCCTCGTGCCAATTGGTTCCCAAATGGCTCATTGCAGATCTCAGGCCTTGTTCCTACAGACCAGGGGAATTACACAGTGCAGTTAGTGACTGCTGAGAGTAGAGCACAACATACAGTTTCCCTGCCAGTTTATG
