# Supplementary material for: Coevolution of paired receptors in Xenopus carcinoembryonic antigen-related cell adhesion molecule families suggests appropriation as pathogen receptors
Source: BMC Genomics. 2016 Nov 16;17:928. doi: 10.1186/s12864-016-3279-9 (PMC5112662; doi:10.1186/s12864-016-3279-9)
Supplement: Additional file 3: — Xenopus laevis ceacam N exon nucleotide sequences. Xenopus laevis ceacam N exon nucleotide sequences (Xenbase laevis 9.1 unless otherwise indicated). EST, expressed sequence tag; N, N or Ig variable-like domain exon; P, pseudogene (stop codon in N exon). (DOCX 22 kb) [file 12864_2016_3279_MOESM3_ESM.docx]

***Xenopus laevis ceacam* N exon nucleotide sequences (Xenbase laevis 9.1 unless otherwise indicated)**

EST, expressed sequence tag; N, N or Ig variable-like domain exon; P, pseudogene (stop codon in N exon)

**L homeologs**

**Group 1**

>Xla_ceacam325N

GCTCGGTATCGTGCCTACAGAATGTATCAAGGAATGAGAGTGAATCTGTAATTTTCACAGTGAAGCTGAATTTACCTGCACAGAATCAGCGGTTGGTAACATGGAGGTTTGGTACCAGTACGATTGCAACTGCAATACAGGGTAACACTCCGACTTATAGTAACAGCTGCACAGACAGATGCTCCCTGTATGGAAACGCTAGTCTCCAGCTGGACAATGTCACTCGTGCAGATACAGGGAACTATACACTCACTGTAACCAACATAGACACTACTGTGCAACAAACAGAACAGTTTCATCTCACGGTTTACG

>Xla_ceacam326N not in Xenbase laevis 9.1; EST EG566377

GTGCTGTAACATCTGCAGAGAATGTAGCTGGGACTGAGGGCAAATCTGTGTATCTCACAGTGAAGTTGGATCTCCCTGCAATGTGGCAGGTAATATGGAGGGTTAATACCAGCACTCAGATTGTAACTGTATCGACTGGCGGATCTCCCACTTATACTGATAGATACAGAAACAGATGCCACCTGTATGATAATACAACTCTACGACTGGACAATCTCACTCACGCAGATACAGGGGAATATTCACTCTCTGTCACCAACCAGAACACTGGAGACACAGTGACAAGATCAGTTTATCTCACAGTTTACA

>Xla_ceacam327N

GCTCAGTATCGTGCCTACAGAATGTATCAAGGAATGAAAGTGAATCTGTAATTTTCACAGTGAAGCTGAATTTACCTGCACAGAATGAGCGGACTGTAACATGGAAGTTTGGTACCAGTCCAATTGCATCTGCAGCACTGGGTAACCCTCCTACTTATAATAACAGCTGCACAGACAGATGCCGCCTGTATGGAAACACTAATCTCCAGCTGGACAATCTCACTCCTGCAGATACAGGGGAATATACAGTCAATGTATTCAACATAATCACTGCTGTGCAACAAACAGAACAGTTTCATCTCACGGTTTACG

>Xla_ceacam328N

GCTCTGTATCTTGCCTACAGAATGTATCAGGGAATGAGGGTGGATCTGTAACTCTCACAGTGAAGCTGAATTTACCTGCACAGAATGAGAGGACCGTAACATGGAAGTTTGGTGTCAGTACAATTGCAACTGCAGTACTGGGTAACCCTCCTACTTATAATAACAGCTGCACAGACAGATGCTCCCTGTATGGAAACGCTAATCTCCAGATGGACAATCTCACTCCTGCAGATACAGGGGAATATACAGTCACTGTATTCAACATACTCACTTCTCAGCAACAAACAGAACAGTTTCATCTCACAGTTTACA

>Xla_ceacam329N

GTGCTGTAACATCTGCAGAGAATGTATCTGGGACTGAGGGCAAATCTGTGTATCTCACAGTGAAGTTGGATCTCCCTGCAATGCGGCAGGTACAATGGAGGGTTAATACCAGCATGATAATTGTAACTGTATTACCTGGCGGCCCTCCTTCTTATACTGATAATTATAGAGGCAGATGCCACCTGTATGATAATACAACTCTCCGACTGGACAATCTCACTCACGCAGACACAGGGGAATATTCACTCTCTGTCACCAACCAGAGCAGCGGAGCCACAGTGACAGGATCAGTTTATCTCACAGTTTACA

>Xla_ceacam330N

GGTCTGTCTCTTGCTCAAGGAATGTATCGGGCACCGTGGGCAAATctgtgactcttccagtgactctgactctgcctGCACAGAGTTCAATACAATGGAAGTTTAAGAATGATTTTATTGTGACTGCGCAGCCGAACGGTATCCCCTTTTATTATGGTGCCTACGAAGGCAGATGCCACCTGTATGAGAACGCAGCTCTCCAGCTGGACAATCTCACTCTCAAAGATACGGGGGAATATACACTCACTGTAATCCCAACAAATGGAGCAACACAAACAGAAGTGTTTTATCTCTCAGTTTACA

>Xla_ceacam331N

GCTCTATATCTTGCCTACAGAATGTATCAGGGAATCAGGGTGGAACTGTAACTCTCACAGTGAAGCTGAATTTACCTGCACAGAATCAGTGGGTGGTAACATGGAGGTTTGGTGTCAGTCCAATTGCATCTGCAGCAGTGGGTAACCCTCCAACTTATAATAACAGCTGCACAGACAGATGCCGCCTGTATGGAAACGCTAATCTCCAGCTGGACAATCTCACTCCTGCAGATACAGGGGAATATACAGTCATTGTAACCAACATAAACGCTGGTCAGCAACAAACAGAACAGTTTCATCTCACAGTTTACA

>Xla_ceacam332N

GTGCTGTAACATCTGCAGAGAATGTAGCTGGGACTGAGGGCAAATCTGTGTATCTCACAGTGAAGTTGGATCTCCCTGCAATGCGGCAGGTACAATGGAGGGTTAATTCCAGCACTCTGATTGTAATTGGATCAACTGGCGGATCTCCTGCTTATACTGATAATTATAGAGGCAGATGCCACCTGTATGATAATACAACTCTACGACTGGACAATCTCACTCATGCAGACACAGGGGAATATTCACTCTCTGTATCCAACCTGGACAGTGGAGCCACAGTGACAGGATCAGTTTATCTCACAGTTTACA

>Xla_ceacam333N

GCTCGGTATCTTGCCTACAGAATGTATCAGGGAATGAGGGTGAATCTGTAATTTTCACAGTGAAGCTGAATTTACCTGCACAGAATCAGCGGGCTGTAACATGGAGGTTTGGTGTCAGTCCAATTGCATCTGCAGCACTGGGTAACCCTCCTACTTATAATAACAGCTGCACAGGCAGATGCTCCCTGTATGGAAACGCTAGTCTTCAACTGGACAATCTCACTCCTAGAGATGAAGGACAATATACACTTCTTATAACCAACATAGACACTGCTCAGCAACAAACAGAACAGTTTCATCTCACAGTTTACA

>Xla_ceacam334N

GGTCTGTATCTGGTTTACTGAGAGAATCAGGAGCTTTGGGAAAATCTGTGAATCTCACAGTGAAGCTGAATCTGCCCACACAGCGTGAAGTACAATGGAAGTTTGGTGCCAACTCTATCATTGCAACTGCCCAGCTGAATAACCCTCCCCTCTATTATGGCAGTTACAGAGGCAGATGCCACCTGTATGATAATACAACTCTCCAGCTGGACAATCTCACTCCTGCAGATACAGGGGTATATTGGCTCTTTGTATCCAACGTGGACACTGCAGCACAACAAAATGGGTCGATTTCTCTTACTGTTTACA

>Xla_ceacam335N1

GGTCTGTCTCTTGCTCAAGGAATGTATCGGGCACTGTGGGCAAATctgtgactcttccagtgactctgactctgcctGCACAGAGGTCGATACAGTGGAAGTTTAATAATGATTTTATTGCGACTGCGCAGCCGAACGGTATCCCCTTTTATTATGGTGCCTACGAAGGCAGATGCCACCTGTATGAGAACGCAGCTCTCCAGCTGGACAATCTCACTCCCGCTGATACGGGGGAATATACACTCTCTGTAATCCCATCAAGTGGAGCAACACAAACAGAAATGATTTATCTCTCAGTTTACA

>Xla_ceacam335N2

GTGCTGTAACATCTGCAGAGAATGTAGCTGGGACTGAGGGCAAATCTGAGTATCTCACAGTGAAGTTGGATCTCCCTGCAATGTGGCAGGTAACATGGAGGGTTAATTCCAGCACTCAGATTGTAACTGTATCGACTGGCGGATCTCCCATTTATACTGATAGATACAGAAACAGAAGCCACCTGTATGATAATACAACTCTCCGACTGGACAATCTCACTCCCACAGATACAGGGGAATTTTCACTCACTGTCACCAACCTGAACAATGGAACCACAATGACAGGATCAGTTTATCTCACAGTTTACA

>Xla_ceacam335N3

GTGCTGTAACATCTGCAGAGAATGTAGCTGGGACTGAGGGCAAATCTGTGTATCTCACAGTGAAGTTGGATCTCCCTGCAATGCGGCTGGTAACATGGAGGGTTAATTCCAGCACTCTGATTGTAACTGCATTGACAGGCGGATCTCCTTCTTATACTGATAGTTACAGAAACAGATGCCACCTGTATGATAATACAACTCTCCGACTGGACAATCTCACTCACGCAGACACAGGGGAATATTCACTCTCTGTCACCAACAACCAGAACACTGGAGTCATACAGACAGGATCAGTTTATCTCACAGTTTACA

>Xla_ceacam338N

GACCTGTATATTGTTTGCTGGAAGAGTCAGGGATTGAGGGCAAATCTGTAAATCTCACAGCGAATCTGAATCTGCCCACACAGCGTGAAGTACAATGGAGGTTTGGTACCAACATTATAATTGCAACTGCCCAGCTAAATAATACTCCCCTCTATTATGGCAGTTACAGAGGCAGATGCCACCTGTATGATAATACAACTCTCCAGCTGGACAATGTCACTCCTGCAGATACAGGGCAATATTCACTATTTGTAACCAACGTGGACACTGCAACACAAAAAAATGGGACGGTTAATCTCACTGTTTACA

>Xla_ceacam339N

GCTCGGTATCTTGCCTACAGAATGTATCAGGGAATGAGGGTGAATCTGTAACTCTCACAGTGAAGTTGAATTTACCTGCACAGAATCAGCGGGTGATAACATGGAGGTTTGGTGTCAGTCCAATTGCATCTGCAATACTGAGTAACCCTCCTACTTATAATAACAGCTGCACAGACAGATGCCGCCTGTATGGAAACATTACTCTTCAGCTGGACAATCTCACTCCTAGAGATGAAGGACAATATACATTTCTTGTAACCAACATAATCACTGGTCAGCAACAAACAGAACAGTTTCATCTCACCATTTACA

>Xla_ceacam340N1

GTGCTGTGTTTTCTATAAAGAATGTGCAGGGTGATGAAAGACAATCTGTGACTCTCAGTGTAAACTTGAGAGTCAACTTGTTTGAAAATCAGCAGATAATGTGGTATTTTAATACAAACACTCCTGTTGCATTTGAAACAACAAACAGCACGCCAAGTTATATTTGGGGGTGTACAGGCAGATGCACCCTGTTTGAGAATGCAACACTCCAGATGGACAATCTGATCCCTGCAAACCAGGGCAATTACACACTCACTATAATGAACTGGGATACAGGATCTACTGTGTCAGGGTCGGTCTATCTCACAGTACAGA

>Xla_ceacam340N2

GAACTTTTTATATACAGCATGTGCAAGGCATTGAAGGTCAATTTGTGACTCTCTGTGTAAAGCTCAATGTGTTTGAAGACCAGATGGCAACATGGTATTTTAATAAATCTAATACGGTTGCATCAGAATCCACGAACAGCACACCAACATATTATTGGGGGTATGATGGGAGATGCACGCTGTTTGAGAATGCAACTCTCCAGCTGGACAGTCTGACCCCTGCAGACCAGGGCAATTACTCCCTTACTATAATGAACTGGAATACAGCATCGTCTGTGTCAGGATGGGTCTATCTTACAATAGAGA

>Xla_ceacam341N GAAATGTGGTTTCTATAAATAATGAGGAGGTTATTGAAGGAGAATCTGTGTCTCTCAGTGTAAAGCTGAACCTATCTGAATATCAGATGATAATATGGAAGTTTGATACATACAATCTGGTTGCATTAGAAACAATGAACAACACTCCAATTTGTTTTCCGGAGTATGAAGGGAGATGCACCTTGTTCGAAAATGCAACGCTGCAGCTGGACAATCTGACCCCTGCAGACCAGGGCAATTACACAGTCACTGTACTGGATGTGGAAACAGGATTGTCCATGTCAGGATCAGTCTATCTCACAGTACTGA

>Xla_ceacam342.L_N

TGACTACTTTGACTGCCCGTCAGTTCGATGGAGCTGTCGGGGGATCTGTGTTTATGTTCACTACCCTGGAGGTGCCGCCACGTAACATAATAACATGGACAGCTGGGAGCAATACGATTGCAACGCTTGAAGATGGTCAAAATCCAACTTACAGCTCAATTTGTGTTTCAAGATGTGAATTGTTTCAAAATGCCACACTGAGGCTGGACAGGCTACTAGTCTCTGATCCCAAATCTTATGACCAAAGCATTAAAAACAAAGATGATGATAAAACTACCTCGATCCCAGTCACTCTCATTGTGCACA

>Xla_ceacam343N1 not in Xenbase laevis 9.1; EST EG581540 GCTCTATATCTTGCCTACAGAATGTATCAGGGAATGAGGGTGAATCTGTAACTCTCACAGTGAAGCTGAATTTACCTGCACAGGATCAGCGGGTGGTAACATGGAGGTTTGGTGACAGTCCAATTGCATCTGCAGCAGTGGGTAACTCACCTACTTATAATAACAGCTGCACAGACAGATGCACCCTGTATGGAAATGCTAATCTTCAACTGGACAATCTCACTCCTAGAGATGAAGGACAATATACACTTCTTGTAACCAACACAGTCACTGCTCAGCAACAAACAGAACAGTTTCATCTCACAGTTTACA

>Xla_ceacam343N2 not in Xenbase laevis 9.1; EST EG581540 GTGCTGTAACATCTGCAGAGAATGTAGCTGGGACTGAGGGCAAATCTGAGTATCTCACAGTGAAGTTGGATCTCCCTGCAATGCGGCTGGTACAATGGAGGGTTAATTCCAGCACTCTGATTGTAAGTGTATCGACTGGCGGATCTCCCACTTATACCGATGCCTACAGAGACAGATGCCACCTGTATGATAATACAACTCTACGACTGGACAATCTCACTCCCGCAGATACAGGGAAATATTCACTCACTGTCACCAACCAGAACAATGGAACCACAGTGACAGGATCAGTTTATCTCACAGTTTACA

**L homeologs**

**Group 2**

>Xla_ceacam376.L_N

TTCTCCTCTCCCTTTTCATGGTTACAGCCAGCAGCCTTACCATTGACTTGGTCCCACAGTGTGCCTTGATCGGCAAGAACGTCACTTTCAGTGTCAGAGGAATAAACGGGAAATTAAACAGCTTTTCCTGGTATCTAGGAAATAATCCAAGTGCTTCAGACCAGATAATTAACTATGTTGTAGGTCTTACACCTCCCACCACTCTTGGACCTAAAAACTTTTCTGATGCCATTGGGCTTCCAAACGGCTCCTTACTGATTACCAACCTCAAGGACCAATACAGTAATAATTACACAGTCCAGGTACAGTCAGATACAGCGAACCAAGCTTCCATAAATCTGTCTGTGAAAC

>Xla_ceacam377N

CATTCATCATCAGTCTTTGGATGGAGTCAAGCTGTGGAATTGATATTCAACTAGTCCCTAGCCATCCACTGATCAATAAGTCTGTCACCTTGAATGTCAGAGGAATCACTGGTACAGTACGAGCCTTCAGGTGGTATTTTGGGTCAAACCCAGATGCTAGCAACCAAATCTTACACTATAATCCAAATTCTAACCCTCCACAAACACCAGGTGATCAGTATTTCCATAGGGCTCATGGGCTCGCAAATGGCTCCTTACATATCTCAGACCTTGTTCGTACAGACCAAGGGAATTACATGGTGATGATATTGGCAAGGGATATCGAAAGAGTGACAGTTTACCTGCCTATTTATG

>Xla_ceacam378N

CTCTCTTCAGTGTCTGGATAAATCTCATCCATGGAATTAAAATCCAGCCAATTCCTGAATATCCAGTAGTCAATCAGCCTGTCACTCTCAGTGTCAGTGATATCAGTGGGACAATTATTTCTTTCTCCTGGTATAAAGGTTTATCAACTGACAATTCGACCCTAATCTTAACATATATTTTGTCTTCTAACCCTGTTGAGACACCAGGGCCACAGCACTTCCCTCAGGCCAGTGGCCTCCCAAATGGCTCATTAAGGATCTCAAAAATTGCAAACAGAAAAATTTACACAGTGCAGATACAGGCAGACAGTTTAACCCAAGCAAGTATTAACCTGCCTGTATATG

>Xla_ceacam379N

CTCTCTTTAGTGTCTGGATAAATCTGATCTCCGGAATCAAAATTCAGCCGATTCCTGAACATCCAGTGATCAATCAGCCTGTCACCCTCAAAGTCAGTGGCGTCAGGGGGGTAATTCGCTCTTTCTCCTGGTATAAAGGTCGATTCGTTGATGATTTCTCCCTAATCTTAACATATGATGCACGTTCTAACACTGTAAAGACACGAGGGCCAGAGTTCTTCCCTCGAGCCAGTGGCCTTCCAGATGGGTCGCTACAGATCTCAAAGCTTTATAAAGCAGTCAGAAGATATTACACAGTTCAAATACAGGCGGACAGTTTGACCCAAGAAACCATTAACCTGCCTGTATATG

>Xla_ceacam380N

CTCTCTTCAGTGTCTGGATTAATCTGATTAATGGAATTAGTATTCAGCTGATGCCTGAATATCCAGTGGACAATCAGCCTGTCACTCTCAGTGTCAGTGGGGTCATTGGTATCATTGAATCTTTCTCCTGGTATAAAAGTTCAACTGTTGTGAATTCCTCATTAATATTAACTTACAATTCATCTTCTTACACGGAGACACAAGGGCCACAGTATTTCTCCCGAGCCAGTGGCCTCCCAGATGGATCATTAAGCATCTCAACCATTTATACTACAGACCAGACGTATTACACCGTGCAGGTAGAGGCAGACAGTTTAACAGTAGACACTATTTACCTGCGTGTTTATG

>Xla_ceacam381N

TTTGCCTCAGTGTTTGGATGATTTCAGCTCATGGAATTGGGGTTCAGCTGATCCCTCAGTATCCGGTGGTTAATCAGTCTGTTACTCTTAGTATCACTGGAGTCACTGGCACAATACGACAGTTCTCATGGTTTAAAGTTTCAAGTGTTCTTGGTTATAGCCAAATATTCAGTGTGAATCCATCTCTAAACACAGCGAAACCAGGGCCTCAGTATTTCCCTCGGGCCAGCTGGTTCCCAAATGGCTCATTGCAGATCTCAGGCCTTGTTCCTATTGACCAGGGGACTTACTCAGTGCAGATATGGGCTTCTAATACATCACATACAACATCAAATACAACTTCAGTTTTCCTGACAGTTTATG

>Xla_ceacam382N

CTCTCTTCAGTGTCTGGATAAATCTGATCCATGGAATCACAATTCAACTGAATCCGAAATATCCAGTAGCCAATCAGCCTGTCACTTTCAGTGTCAGTGGATTCAGGGGGACGGTTCTTTCTTTCTCCTGGTACAGAGGTTTACCAGTTGACAATTCCTGCCTAATCTTAACTTATAATTCATCTTCTAATCCTAAAGAAACACGAGGGCAACGATACTCTTCTCAGTTCAGCGTCCTTCCAGATGGATCATTAAAGATCTCAAGCCTTTCTTCTTCATACAGAGATTATTACACAGTGCAAGTACAGGCAGAAAGTTTAACCCAAAAAAGTATTTACCCGCAGTACTATG

>Xla_ceacam383N

CTCTCTTCAGTGTCTGGATAAATCTGATCTCCGGAATCAAAATTAAGCCGATTCCTGAATATCCAGTAGTCAATCAGCCTGTCACCCTCAAAGTCAATGGGGTGAGAGGGACAATTCGCTCTATCTCCTGGTATAAAGGTCGATCTGTTGACAGATCCTTCCTAATCTTATCATATAATGCCACTTACAATGCAAAGACACAAGGGCCAGAGTTCTTTCCTCAAGCTAAAGTTCTCTCAGATGGATCATTGAGCATCTCTGGACTTTATAAAGCAGTCAGAAGATATTACACAGTGCAAATAGAAGCAGAAAGTTTAACCCAAGAAAGTATTGACCTGCCTGTATATG

>Xla_ceacam384N

CTCTCTTCAGTGTCTGGATAAATCTGATCCATGGAATCAAAATCCAGCCAATTCCTGAATATCCAGTAGTCAATCAGCCTGTCACTCTCAGTGTCAGTGATATCAGTGGGACAATTATCTCTTTCTCCTGGTATAAAGGTTCATCCATCAGCAATTCCTACCTAATCTTAACTTATAATTCAACTTCTAACCCTGTGCAGACACCAGGGCCACAATACATCCCTCATTCCAGTGGCCTCCCAAATGGCTCATTACATATATTGGAACTTTCTACTTCAAACAGAGGAAATTACATAGTGCAAATACAGCCAGGAAGTCAAACATCACAATATATTAACCTGCCGGTATATG

>Xla_ceacam385N

TTTGCCTCAGTGTTTGGATGGATTCAGCCCATGGAATTGGGGTTCAGCTGATCCCTCAGTATCCGGTGGTTAATCAGTCTGTTACCCTGAGTGTCACTGGAGTCACTGGCACAATACGAGTATTCGCATGGTATAAAGGTTCAAGTGTAGATACTAATAACCAAATATTCAATGTTATTCCATCTCTAAATTCAGTGACACATGGGCGTCAGTATTTCTCTAGGGCCAGTCAGTTCCCAAATGGCTCATTGCAGATCTCAGGCCTCGTTCCTACAGACCAGGGGAATTACACAGTGTTTATACAGACTGCTGAGCATACAGAACAACATACAGTTCTCCTGACAGTTTATG

>Xla_ceacam386N

TTTGCCTCAGTGTTTGGATGGATTCAGCCCATGGAATTGGGGTTCAGCTGATCCCTCAGTATCCGGTGGTTAATCAGTCTGTTACCCTGAGTGTCACTGGAGTCACTGGCACAATAAGAGAGTTCACATGGTATAAAGGTTCAGTTGCAGCTGCTCCTAATCAAATTTTCAATGTTACTCCATCTCTAAACTCAACAAATGGGACTCAGTATTTCCCTCAGGCCAAAGGGTTCCGGAATGGCTCATTGCAGATTTCAGGCCTTGTTCCATCAGACCGGGGGAATTACGTAGTGCAGGTACAGACTGGGAGTGTAACACAAGTGACAGTTTTCCTGCCAGTTTATG

>Xla_ceacam387N

TTTGCCTCAGTGTGTGGATGGATTCAGCCCATGGAATTGGGGTTCAGCTGATCCCTCAGTATCCGATGGTTCGTCAGTCTGTTACCCTGAGTGTCACTGGAGTCACTGGCACAATACGACAGTTCACATGGTATAAAGGTTCAAGTACAGATGATAATAACCAAATATTTAATGTTATTCCAGAAACAAACTCAGTGACGAATGGGCCTCAGTATTTCTCTAGGGCCAGTCATTTCCCAAATGGCTCATTGCAGATCTCAGGCCTCGTTCCTACAGACCAGGGGAATTACACAGTGTTTATACAGACTATAGAGGGTACAGCACAACATACAGTTCTCCTGACAGTTTATG

>Xla_ceacam388N

TTTGCCTCAGTGTTTGGATGGATTCAGCCCATGGAATTGGGGTTCAGCTGATCCCTCAGTATCCGGTGGTTAGTCAGTCTGTTACCCTGAGTGTCACTGGAGTCACTGGCACAATACGACAGTTCTCATGGTATAAAGGTTCAAGTGTAGATGCTAATAACCAAATATTCAATGTTATTCCATCTCTAAACTCAGTGACAAATGGGCGTCAGTATTTCCCTCGGGCCAGTCATTTCCCAAATGGCTCATTGCAGATCTCAGGCCTTGTTCCTACAGACCAGGGGAATTACACAGTGTTTATACAGACTGGTGAGACTACAGCACAACATACAGTTCTCCTGACAGTTGATG

>Xla_ceacam389.L_N

TTTGCCTCAGTGTTTGGATGGATTCAGCCCATGGAATTGGGGTTCAGCTGATCCCTCAGAATCCGGTGGTTAATCAGTCTGTTACCCTGAGTGTCACTGGAGTCACTGTTACAATACGACAGTTCTCGTGGTATAAAGGTTCAAGTGTAGATACTAATAACCAAATATTCAATGTTATTCCACAAACAAACTCAGTGACACATGGGCGTCAGTATTTCTCTAGGGCCAGTCATTTCCCAAACGGCTCATTGCAGATCTCAGGCCTTGTTCCTACAGACCAGGGGAATTACACAGTGTTGATACAGACTATAGAGACTTCAGCACAACATACAGTTCTCCTGACAGTTTATG

>Xla_ceacam390.L_N

CTCTCCTCAGCTTTTTGATGGACTCAACAAGAGCCATCACCATCGAGGTGATCCCTAAACACCCAACTCCCAATTCGAATGTGATTCTCAGTGTTAAAGATGTCGCCGGTGAAATAAGAAGCTTCAGCTGGTACAATGGATCAAACCCAAGTGCTTCAAATCAAATCTTAAACTATATCCCATCTCTTACGCCACCACAGACCAAAGGACATATGTATTTTACTGAAGCCGAGGGACTGGCAAATGGCTCCCTTCTGATCAAAGACTTTGTGAAGAAGTTTGAGGGTGTGTACACAGTGCAGATACAAGCGGAGAGCCCATTACAAGCATCAGTAACAGTAACCATGAGTG

**S homeologs**

**Group 1**

>Xla_ceacam342.S

TGACTACTTTGACTGCCCGTCATTTCGATGGAGCTGTCGGGGGATCTGTGTTCATGTTCACTTACCTGGACGTACCGCCACATTACGTGATAACATGGACAACTGGTAGCACTACCCTTGCAAGTCTTGAAGATGGCAAAAGTCCCACTTACGGCTCAATTTGTGCTACAAGATGTGAATTGTTTCAAAATGCAACTCTGAGGCTGGATAGCCTAATGAGCAATGATCCCAAAAGTTATATGCAAAGCATTAAAAACAAAGCTAACGATCTCACTACCTCCATTCCAGTCACTCTCCATGTGCACA

**S homeologs**

**Group 2**

>Xla_ ceacam376.1.S_N

TTTTCCTCTCTCTGTTCATGGTTGCAGCCAGCAGCCTCACCATTGAGTTGATCCCACAGTGTGCCTTGATCGGCAAGAACGTCACTCTCAGTGTCAGTGAAATAAACGGGATGTTAAAAAGCTTTTCCTGGTATCTAGGAGATGCTCCAAGTGCTTCAAACCAGATAATTAACTACCTTGTAGGGCTTACACCTCCCACCACTAATGGACCCAAACACTTTACTGATGCCATTGGACTTCCAAACGGTTCATTACTGATTACAAACCTGAAGGAAGAATACAGTAATACCTACACAGTCCAGGTACAGGCAGATTCACCGGACCGAGCTTCAGCTGACCTGATTGTGACAA

>Xla_ceacam376.2.S_N

TGCTCCTCTCTCTGTTCATGGTTGCAGCCAGCAGCCTCACCATTGAGTTGATCCCACAGTGTGCCTTGAAGGGCAAGAACGTCACTCTCAGTGTCAGTGAAATAAACGGGATGTTAAAAAGCTTTTCCTGGTATCTAGGAGATGCTCCAAGTGCTTCAAACCAGATAATTAACTACCTTGTAGGGCTTACACCTCCCACCACTAATGGACCCAAAAATTTTTCTGATGCCATTGGACTTCCAAACGGTTCCTTATTGATTGCAAACCTGAAGGATGAATACAGTAATACCTACACAGTCCAGGTACAGGCAGATTCACCGGACCAAGTTACAAAAAAACTGACTGTGACAA

>Xla_ceacam389.S_N

TTTGCCTCAGTGTTTGGATGGATTCAGCCCATGGAATTGGGGTTCAGCTGATCCCTCAGAATCCGGTGGTTAATCAGTCTGTTACCCTGAGTGTCACTGGAGTCACTGGCACAATACGACTGTTCACATGGTATAAAGGTTCAAGTACAGATGCTAATGTCCAAATATTCAGTGTTATTCCATCTGTAAACTCAGTGACAAATGGGCTTCAGTATTTCCCTCGGGCCAGTCAGTTCCCAAATGGCTCACTGCAGATCTCAGGCCTGGTTCCTACAGACCAGGGGAATTACACAGTGTTTATACAGACTATAGAAAGTACAGCTCAACATACAGTTCTCCTGACAGTTTATG

>Xla_ceacam390.S_N

CTCTCCTCAGCTTGTTGATGGACACGACAAGAGGAATCACCATTGAAGTGATCCCTACACGTCCAATTCCCAATTCAGATGTTACTCTCAAAGTCAAAGGTGTCATTGGGACAATAAGAAGCTTCAGCTGGTACAATGGATCCAACCCAAGCGCTTCAAATCAAATCTTAAACTATATCCCATCTCTTACACCACCACAGACCAAAGGACATATGTACTTCGATCAGGCAGAAGCACTGGCAAATGGCTCACTTCTGATCAAAAACTTCGTGAAGACATTTGAGGGTTTTTACACAGTGCAGATACAAGCAGAGAGCCCACTACAAGCATCGGTGTCTGTAACTATGAGTG
