# Supplementary material for: Coevolution of paired receptors in Xenopus carcinoembryonic antigen-related cell adhesion molecule families suggests appropriation as pathogen receptors
Source: BMC Genomics. 2016 Nov 16;17:928. doi: 10.1186/s12864-016-3279-9 (PMC5112662; doi:10.1186/s12864-016-3279-9)
Supplement: Additional file 4: — Modeling of the three-dimensional structure of Ceacam301 and Ceacam350 IgV-like domains. Ceacam301 and Ceacam350 mature IgV-like domains were modeled using the geno3D software. The equivalent surface corresponding to the CC’C”FG face is shown as ribbons. The amino acids which flank the CC’C” and FG β-strands are indicated in three-letter code. Amino acids which belong to the CC’C”FG face of the sequence alignment are indicated in yellow. Human and murine CEACAM1 IgV-like domain sequences were used as template for modeling of Ceacam301 and Ceacam350 IgV-like domains, respectively. Note the overall similarity of the putative ligand-binding region shown at the left side despite the fact that the regions corresponding to the C” β-strand in CEACAM1 were not classified as β-strands in Ceacam301 and Ceacam350. (PPTX 190 kb) [file 12864_2016_3279_MOESM4_ESM.pptx]

## Slide 1
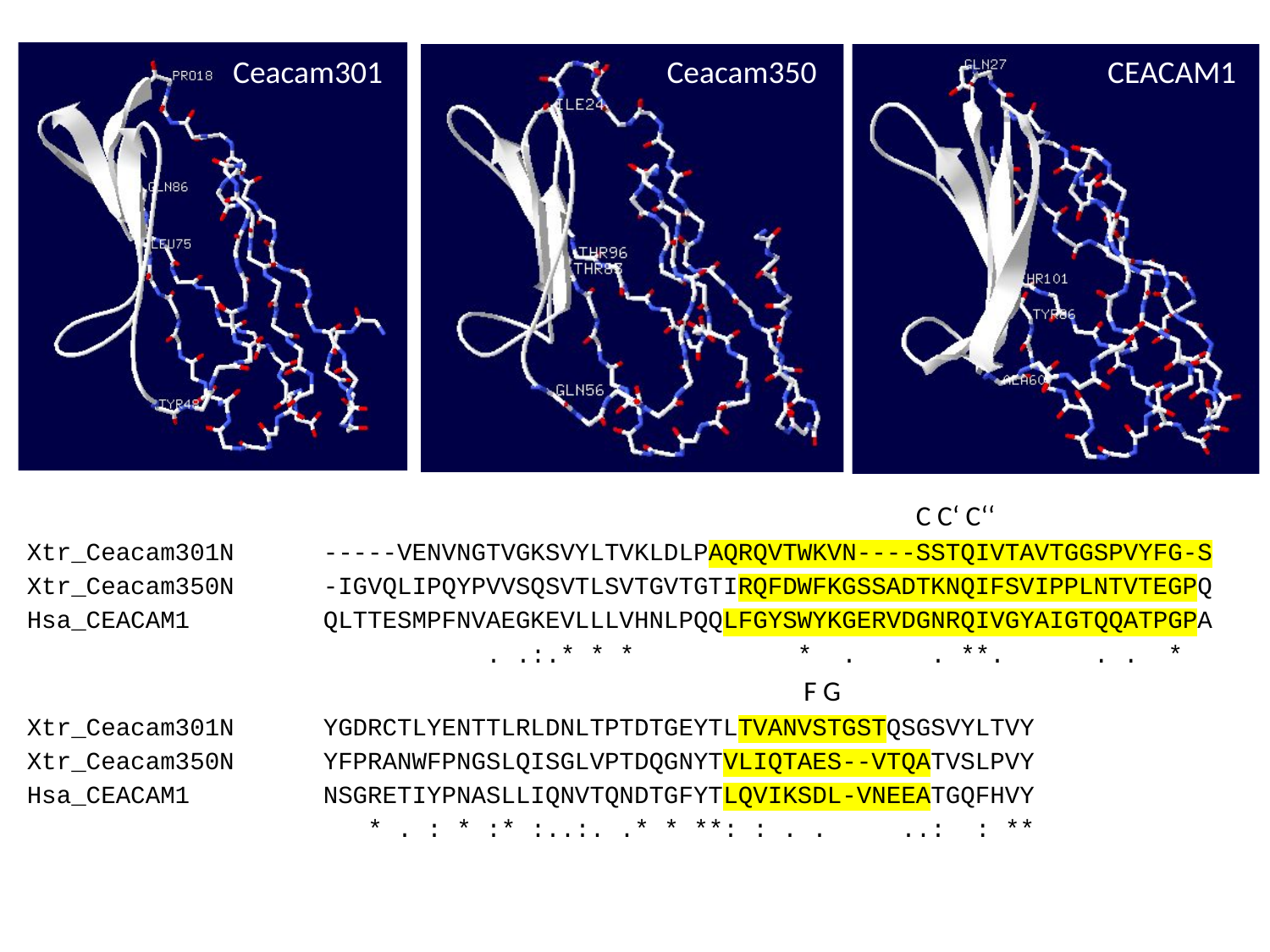

Ceacam350
CEACAM1
Ceacam301
							C C‘ C‘‘
Xtr_Ceacam301N -----VENVNGTVGKSVYLTVKLDLPAQRQVTWKVN----SSTQIVTAVTGGSPVYFG-S
Xtr_Ceacam350N -IGVQLIPQYPVVSQSVTLSVTGVTGTIRQFDWFKGSSADTKNQIFSVIPPLNTVTEGPQ
Hsa_CEACAM1 QLTTESMPFNVAEGKEVLLLVHNLPQQLFGYSWYKGERVDGNRQIVGYAIGTQQATPGPA
 . .:.* * * * . . **. . . *
 						 F G
Xtr_Ceacam301N YGDRCTLYENTTLRLDNLTPTDTGEYTLTVANVSTGSTQSGSVYLTVY
Xtr_Ceacam350N YFPRANWFPNGSLQISGLVPTDQGNYTVLIQTAES--VTQATVSLPVY
Hsa_CEACAM1 NSGRETIYPNASLLIQNVTQNDTGFYTLQVIKSDL-VNEEATGQFHVY
 * . : * :* :..:. .* * **: : . . ..: : **
