# Supplementary material for: Coevolution of paired receptors in Xenopus carcinoembryonic antigen-related cell adhesion molecule families suggests appropriation as pathogen receptors
Source: BMC Genomics. 2016 Nov 16;17:928. doi: 10.1186/s12864-016-3279-9 (PMC5112662; doi:10.1186/s12864-016-3279-9)
Supplement: Additional file 5: — Sequence comparison of leader and N exon genomic regions from Xenopus paired receptor ceacam genes. Nucleotide sequences comprising N domain exons and flanking regions from presumed paired receptor ceacam genes (group 1 and group 2) as well as from the homeologous group 2 inhibitory receptor genes from X. laevis were pairwise aligned. Identical nucleotide positions are shown in red. Splice acceptor and donor consensus sequences are marked in yellow and blue, respectively, translational start codons in green. Note extended stretches of nucleotide sequences with high conservation restricted to N exon regions including splice sites but extending barely in to the introns.Xla, X. laevis; Xtr, X. tropicalis. (DOCX 83 kb) [file 12864_2016_3279_MOESM5_ESM.docx]

Group 1 ceacam genes

*X. tropicalis*

10 20 30 40 50 60 70 80 90 100 110 120 130 140 150

**leader exon**

| | | | | | | | | | | | | |

**start codon**

**splice donor**

Xtr_ceacam301 ---------ATGGGAGCCTGCTGGTACCTGTCCGTTTGGGTCCTGCTGCTCCTGGCT--------GTCTCAG**GTAAGG**GCT---TACAAGAAAA---TACAGAA----ATACAAATGA-GCAATCGTTAGCTGGCTG---GAAAATAAAT

Xtr_ceacam303 ATGGAGCACTTTGTTCTCCCTCAGGGTCTTTCCCTCTGTATCCTGCTCCTCTTGGCTTTAG**GTGAGT**CCCTGGTACCAGATAAATACATTAATACTGTACATGGTATCATACATATACTGTAATATAGGACTTGCTGTGTGAGAGTAAGT

160 170 180 190 200 210 220 230 240 250 260 270 280 290 300

| | | | | | | | | | | | | |

**splice acceptor**

Xtr_ceacam301 G--------TTTAGGCGTCCTGTATGGA------------CCCGACAATT------------AAACA---AATCGCCTAGTGATTGCAGCTGTGATTTCTATTCCCAC**AG**GTACTGTAAGATCTGTAGAGAATGTAAATGGGACTGTGGG

Xtr_ceacam303 GCACTGCTATTCAGGATTTGTTTATGAATGGGATATAGTACCTGAAAGTTTACATGTTTTGGAAACACTGACCTCTCTGATGATAAGTTTCCTTCTATTTGCTTCCAC**AG**GTACTGTAAGATCTGTAGAGAATGTAACTGGGACTGTGGG

310 320 330 340 350 360 370 380 390 400 410 420 430 440 450

**N exon**

| | | | | | | | | | | | | | |

Xtr_ceacam301 CAAATCTGTGTATCTCACAGTGAAGTTGGATCTCCCGGCACAGAGGCAGGTAACATGGAAGGTTAATTCCAGCACGCAGATTGTAACTGCAGTAACTGGAGGCTCTCCCGTTTATTTTGGAAGCTACGGAGACAGATGCACCCTGTATGA

Xtr_ceacam303 CAAATCTGTGTATCTCACAGTGAAGTTGGATCTCCCGGCACAGAGGCAGGTAACATGGAAGGTTAATTCCAGCACGCAGATTGTAACTGCAGTAACTGGAGGCTCTCCCGTTTATTTTGGAGACTACGGAGGCAGATGCACCCTGTATGA

460 470 480 490 500 510 520 530 540 550 560 570 580 590 600

| | | | | | | | | | | | | |

**splice donor**

Xtr_ceacam301 GAACACAACTCTCCGGCTGGACAATCTCACCCCCACAGATACAGGGGAATATACACTCACTGTAGCCAACGTGAGCACTGGATCAACACAATCAGGATCAGTTTATCTCACAGTTTACA**GTAAGT**GTATCGAACCTATTAAATACATGGA

Xtr_ceacam303 GAACACAACTCTCCGGCTGGACAATCTCACTCCCACAGATACAGGGGAATATACACTCACTGTAGCCAACACGGGCACTGGAGCAACACAATCAGGATCAGTTTATCTCACAGTTAACA**GTAAGT**GGGTTTTTCAATTCCTTTCTCTGGC

610 620 630 640 650 660 670 680 690 700 710 720 730 740 750

| | | | | | | | | | | | | | |

Xtr_ceacam301 ATAATAATTGCAAAAGTCTATTTTAAAGCAAGGCTAGAAGATAACATACACATGAAAAGATCAT-CAGTACAGGACAGAAAAGGGCACCCTTATCCTAACACAATGGGATCCATTCATTCAAGTCCATCCATTCATTCAAGTCAATATTT

Xtr_ceacam303 TTTGGCTCTGCCAGTAAAAACTCTGCTGAGAGGATATCAAATCACTGTTACCAGCAGGCATGTTGCAGTTTCAAAAGTTAAAAATCTAAATGCCCAACATGTTTTGTGCGCATATAATTCCAAAACACACACACTTAGGGGCTGAT-TTA

*X. laevis*

10 20 30 40 50 60 70 80 90 100 110 120 130 140 150

**splice donor**

| | | | | | | | | | | | | | |

**start codon**

**leader exon**

Xla_CEACAM325 ATGGGACCC-TGTAACCTGCTGCTGCTGCTGGTGACC-----GCTATAGGTA-AGGGCTACTTCT----------------GAAGAGAGAGGG-CAGAGATCGATGTTCGTAAATTT----------------AGGGACGCTTCACGATC

Xla_ceacam328 CTGGCACCTGTCTGCCCCAATACTGCTGCTGTTCTCATACGGGACACAGACATAACATTATTCTTTCATAATGACCTAAATGAGGCAAGAGGAACAGAGAGCAATGTCCATTCATTTCATGAATAATTTGTAGAAAGACACATCAGGCAC

160 170 180 190 200 210 220 230 240 250 260 270 280 290 300

| | | | | | | | | | | | | |

**splice acceptor**

**N exon**

Xla_CEACAM325 ACGACCTAATAATGTGTC--TTA---------TGTTCCCCCCGCAGGCTCGGTATCGTGCCTACAGAATGTATCAAGGAATGAGAGTGAATCTGTAATTTTCACAGTGAAGCTGAATTTACCTGCACAGAATCAGCGGTTGGTAACATGG

Xla_ceacam328 AAGAAATAACAATTTGTTCATTAAAACGTTGCTTTTCCTCTCACAGGCTCTGTATCTTGCCTACAGAATGTATCAGGGAATGAGGGTGGATCTGTAACTCTCACAGTGAAGCTGAATTTACCTGCACAGAATGAGAGGACCGTAACATGG

310 320 330 340 350 360 370 380 390 400 410 420 430 440 450

| | | | | | | | | | | | | | |

Xla_CEACAM325 AGGTTTGGTACCAGTACGATTGCAACTGCAATACAGGGTAACACTCCGACTTATAGTAACAGCTGCACAGACAGATGCTCCCTGTATGGAAACGCTAGTCTCCAGCTGGACAATGTCACTCGTGCAGATACAGGGAACTATACACTCACT

Xla_ceacam328 AAGTTTGGTGTCAGTACAATTGCAACTGCAGTACTGGGTAACCCTCCTACTTATAATAACAGCTGCACAGACAGATGCTCCCTGTATGGAAACGCTAATCTCCAGATGGACAATCTCACTCCTGCAGATACAGGGGAATATACAGTCACT

460 470 480 490 500 510

**splice donor**

| | | | |

Xla_CEACAM325 GTAACCAACATAGACACTACTGTGCAACAAACAGAACAGTTTCATCTCACGGTTTACGGTAAGTACCGC

Xla_ceacam328 GTATTCAACATACTCACTTCTCAGCAACAAACAGAACAGTTTCATCTCACAGTTTACAGTAAGTACTGA

Group 2 ceacam genes

*X. tropicalis*

10 20 30 40 50 60 70 80 90 100 110 120 130 140 150

| | | | | | | | | | | | | |

**splice acceptor**

Xtr_ceacam350 GCTTCAAGTGTACCCCAGATATTTTCTAATATATTCAT---------TTTTCTCTTTC**AG**TTTCCCTCAGTGCTTGGATGGATGGAGCCCATGGAATTGGGGTTCAGCTGATCCCTCAGTATCCGGTGGTCAGTCAGTCTGTTACTCTGA

Xtr_ceacam351 CTTGCCCCTGTGCCTCAGTTCTCCCCTTCTCTACTGATCTGGCTTTATTCTCTCTTTC**AG**TTTCCCTCAGTGCTTGGATGGATGGAGCCCATGGAATTGGGGATCAGCTGATCCCTCAGTATCCGGTGGTTAGTCAGTCTGTTACCCTGA

160 170 180 190 200 210 220 230 240 250 260 270 280 290 300

**N exon**

| | | | | | | | | | | | | | |

Xtr_ceacam350 GTGTCACTGGGGTCACTGGCACCATACGGCAGTTCGACTGGTTTAAAGGTTCAAGTGCAGATACCAAAAACCAAATATTCAGTGTTATTCCACCTTTAAACACAGTGACAGAAGGGCCTCAGTATTTCCCTCGTGCCAATTGGTTCCCAA

Xtr_ceacam351 GTGTCACTGGGGTCACTGGCACAGTAATCGCATTCGCATGGTATAAAGGTTCAAGTGCAGATACCAATAACCAAATATTCATTGTTATTCCATCTTTAAACTCAGTGACAAAAGGGCCTCAGTATTTCCCTCGTGCCAATTGGTTCCCAA

310 320 330 340 350 360 370 380 390 400 410 420 430 440 450

**splice donor**

| | | | | | | | | | | | | | |

Xtr_ceacam350 ATGGCTCATTGCAGATCTCAGGCCTTGTTCCTACAGACCAGGGGAATTACACAGTGCTGATACAGACTGCTGAGAGTGTAACACAAGCTACAGTTTCCCTGCCAGTTTATG**GTGAGT**AATGTATGATACAGCTAATTTACTGAACTAGTG

Xtr_ceacam351 ATGGCTCATTGCAGATCTCAGGCCTTGTTCCTACAGACCAGGGGAGTTACACAGTGCTGATAGAGACTGCTGAGAGTAGAGCACAACATACAGTTTCCCTGCCAGTTTATG**GTGAGT**AACACGAGGCTTTATGGCCGTACCGTACTCTAC

460 470 480 490 500 510 520 530 540 550 560 570 580 590 600

| | | | | | | | | | | | | | |

Xtr_ceacam350 CAAAGGTACACTGATGCTGCCACCTTCGGGGCCCCCCACCCCAGTCACAGACTTCTAAATGTTCGCACCTCCTCCAACCCCCCCTGCACGCATGTACGATGTG-CTGCACGCCGTGTTATGTTCT--TAGGGGGTGAGGGCCTAAGTTGC

Xtr_ceacam351 TGAA---A--CCAATGTTACC--CTACTGGTGCTGCCACTTTCCCTTCTTGCTAGCAACTGTGTGTACATCA---GGGTGTCTCAGTA-GTTTTCTCATTGTGACTGAGCCACCTATTAAAACCAAGTAATACTTGACCCTTTCAGTGCC

10 20 30 40 50 60 70 80 90 100 110 120 130 140 150

| | | | | | | | | | | | | |

**splice acceptor**

Xtr_ceacam350 GCTTCAAGTGTACCCCAGATATTTTCTAATATATTCAT---------TTTTCTCTTTC**AG**TTTCCCTCAGTGCTTGGATGGATGGAGCCCATGGAATTGGGGTTCAGCTGATCCCTCAGTATCCGGTGGTCAGTCAGTCTGTTACTCTGA

Xtr_ceacam368 CTTGCCCCTGTGCCTCAGTTCTCCCTTTCTCTACTGATCTGGCTTTATTCTCTCTTTC**AG**TTTCCCTCAGTGCTTGGATGGATGGAGCCCATGGAATTGGGGTTCAGCTGATCCCTCAGTACCCGGTGGTTAATCAGTCTGTTACCCTGA

160 170 180 190 200 210 220 230 240 250 260 270 280 290 300

| | | | | | | | | | | | | | |

**N exon**

Xtr_ceacam350 GTGTCACTGGGGTCACTGGCACCATACGGCAGTTCGACTGGTTTAAAGGTTCAAGTGCAGATACCAAAAACCAAATATTCAGTGTTATTCCACCTTTAAACACAGTGACAGAAGGGCCTCAGTATTTCCCTCGTGCCAATTGGTTCCCAA

Xtr_ceacam368 GTGTCACTGGGGTCACTGGCACAATACGGCAGTTCAGCTGGTATAAAGGTTCAAGTACAGATACCAATAACAATATATTCAATGTTATTCCATCTGCAAACTCAGTGACACCAGGGGCTCAGTATTTCCTTCGTGCCAATTGGTTCCCAA

310 320 330 340 350 360 370 380 390 400 410 420 430 440 450

**splice donor**

| | | | | | | | | | | | | | |

Xtr_ceacam350 ATGGCTCATTGCAGATCTCAGGCCTTGTTCCTACAGACCAGGGGAATTACACAGTGCTGATACAGACTGCTGAGAGTGTAACACAAGCTACAGTTTCCCTGCCAGTTTATG**GTGAGT**AATGTATGA---TACAGC--TAATTTACTGAAC

Xtr_ceacam368 ATGGCTCATTGCAGATCTCAGGCCTTGTTCCTACAGACCAGGGGAATTACACAGTGCTGATAGTGACTGCTGAGAGTGTAACACAAGCTACAGTTTCCCTGCCAGTTTATG**GTGAGT**AACACATTGCTTTATGGCCGTACCATACTCTAC

460 470 480 490 500 510 520 530 540 550 560 570 580 590 600

| | | | | | | | | | | | | | |

Xtr_ceacam350 TAGTGCAAAGGTA----CACTGATGCTGCCACCTTCGGGGCCCCCCACC-CCAGTCACAG-----ACTTCTAAATGTTCGCACCTCCTCCAACCCCCCCTGCACGCATGTACGATGTGCTGCACGCC--GTGTTA--TGTTCTTAGGGGG

Xtr_ceacam368 TGAAACCAATGTTACCCTACTGGTGCTGCCACTTTCACTTTCCCTTCTTGCTAGCAACTGTTTGTACATCAGGGTGTCGTTTTCTCGTTGTGACTGAGCCACTTATTAAAACCAAGAAATGCCCTTTCAGTGCCAGCTAATTTTCACACT

Evidence for gene conversion between *ceacam* homeologs in L/S ceacam loci in *X. laevis*

10 20 30 40 50 60 70 80 90 100 110 120 130 140 150

| | | | | | | | | | | | | | |

Xla_ceacam389.L CACACACCGAACAAGAAAATGTGTATAA--TTGCCTTGTGTGCCGTTATACTAAGGT-TATTATTTAATATAAA--TATGAATATACTGTCACCCATGGACAGGGGCATTCAGTCATTTCTGTATGTTATAATACTGTATGTGCTGCAAA

Xla_ceacam389.S AGGACTCTACAGTAGAACCCATATTTTACTTTTCGCAAGGAAACGGGACAAAATGGTGTGAAGGAAAATCTAAAATCAGGGACAGGTTATAAAT--TGGAGGGCTGCAAAAAAAAAATGTAAAATGTGGGAAAACTTAAAATCATGGTAT

160 170 180 190 200 210 220 230 240 250 260 270 280 290 300

| | | | | | | | | | | | | |

**splice acceptor**

Xla_ceacam389.L GTTGCAACTCCTGATATTTTCTAATATATTTATTTCTCTCTTTC**AG**TTTGCCTCAGTGTTTGGATGGATTCAGCCCATGGAATTGGGGTTCAGCTGATCCCTCAGAATCCGGTGGTTAATCAGTCTGTTACCCTGAGTGTCACTGGAGTC

Xla_ceacam389.S GTAA-AATTGAGATTTCATTATAATATATTAATTTCTCTCTTTC**AG**TTTGCCTCAGTGTTTGGATGGATTCAGCCCATGGAATTGGGGTTCAGCTGATCCCTCAGAATCCGGTGGTTAATCAGTCTGTTACCCTGAGTGTCACTGGAGTC

310 320 330 340 350 360 370 380 390 400 410 420 430 440 450

**N exon**

| | | | | | | | | | | | | | |

Xla_ceacam389.L ACTGTTACAATACGACAGTTCTCGTGGTATAAAGGTTCAAGTGTAGATACTAATAACCAAATATTCAATGTTATTCCACAAACAAACTCAGTGACACATGGGCGTCAGTATTTCTCTAGGGCCAGTCATTTCCCAAACGGCTCATTGCAG

Xla_ceacam389.S ACTGGCACAATACGACTGTTCACATGGTATAAAGGTTCAAGTACAGATGCTAATGTCCAAATATTCAGTGTTATTCCATCTGTAAACTCAGTGACAAATGGGCTTCAGTATTTCCCTCGGGCCAGTCAGTTCCCAAATGGCTCACTGCAG

460 470 480 490 500 510 520 530 540 550 560 570 580 590 600

**splice donor**

| | | | | | | | | | | | | |

Xla_ceacam389.L ATCTCAGGCCTTGTTCCTACAGACCAGGGGAATTACACAGTGTTGATACAGACTATAGAGACTTCAGCACAACATACAGTTCTCCTGACAGTTTATG**GTGAGT**ATACAGTGTGCTCACGTCATACAGCTTATTTATTAGTGTAA---AAG

Xla_ceacam389.S ATCTCAGGCCTGGTTCCTACAGACCAGGGGAATTACACAGTGTTTATACAGACTATAGAAAGTACAGCTCAACATACAGTTCTCCTGACAGTTTATG**GTGAGT**A----ATCTGTGCACTTTATACAGCTCAATTACTGAAGTAATGCATG

610 620 630 640 650 660 670 680 690 700 710 720 730 740 750

| | | | | | | | | | | | | | |

Xla_ceacam389.L TCTACTGTTCATTTGTGTACAGCATTGGGGTGGATTCAGTTTTACACTTTAGACTTGCCCCTGCCTTTCTATATAAATTCTTGT--TTGTAATATAATTTAGGATTGAATTACTAATGCAAGGTACAGTGTAAACCAAACAATTGTTTCC

Xla_ceacam389.S TTTATGGAACCATTGTAAGCAGCATTTGGATAGATGCAATTTTACACTTTACACTCACTT-TGTCTTTGTATATAAGCTGTTATATCTGTAATATAATTTAGGACTTAATAATTTTTTTTTTTTTTTGCCTTCTGCTCTCTTTCTTTTCA
